# In some professions, women have become well represented, yet gender bias persists—Perpetuated by those who think it is not happening

**DOI:** 10.1126/sciadv.aba7814

**Published:** 2020-06-26

**Authors:** C. T. Begeny, M. K. Ryan, C. A. Moss-Racusin, G. Ravetz

**Affiliations:** 1 Department of Psychology, University of Exeter, Exeter, England, UK.; 2 Faculty of Economics and Business, University of Groningen, Groningen, the Netherlands.; 3 Department of Psychology, Skidmore College, Saratoga Springs, New York, USA.; 4 British Veterinary Association, London, England, UK.; 5 Simplyhealth, Andover, England, UK.

## Abstract

In efforts to promote equality and combat gender bias, traditionally male-occupied professions are investing resources into hiring more women. Looking forward, if women do become well represented in a profession, does this mean equality has been achieved? Are issues of bias resolved? Two studies including a randomized double-blind experiment demonstrate that biases persist even when women become well represented (evinced in veterinary medicine). Evidence included managers evaluating an employee randomly assigned a male (versus female) name as more competent and advising a $3475.00 higher salary, equating to an 8% pay gap. Importantly, those who thought bias was not happening in their field were the key drivers of it—a “high risk” group (including men and women) that, as shown, can be readily identified/assessed. Thus, as other professions make gains in women’s representation, it is vital to recognize that discrimination can persist—perpetuated by those who think it is not happening.

## Teaser

If you think gender discrimination is no longer an issue in your profession, you may be perpetuating it.

## INTRODUCTION

Women remain underrepresented in a number of professions, including certain fields of science, technology, engineering, mathematics, and medicine (STEMM) (*1*–*3*). Evidence also indicates that women who work in these male-dominated fields are prone to experiencing bias and discrimination. This bias can be expressed by both men and women and can have multiple adverse implications (e.g., for women’s pay and promotion, performance evaluations, and treatment among colleagues) (*4*–*13*).

However, efforts are underway in many of these fields to increase the representation of women (*14*–*16*). In part, this has meant working to address the so-called pipeline problem—the idea that in some professions, one of the obstacles to gender equality is a lack of women pursuing degrees and ultimately careers in them. Such efforts to increase women’s representation may be motivated partly by a belief that once enough women enter the profession, broader issues of bias and inequality will subside (e.g., because having more women in the profession “will naturally lead to a more inclusive culture”) (*17*). Following from this, if someone sees women become well represented in a profession (e.g., biological sciences and veterinary medicine), they may infer that the profession has indeed become more equitable—that the biases and differential treatment that once disadvantaged women are no longer an issue.

In the current research, we examine the veracity of this idea. We test whether gender bias (differential evaluations and treatment of women relative to men) is now absent or remains evident in a profession once dominated by men but now with a substantial representation of women. While existing evidence suggests that gender bias persists in professions still comprised mostly of men (*4*, *7*), there is very little evidence—and none to our knowledge that comes from randomized double-blind experimental data coupled with large-scale, highly ecologically valid field survey data—indicating whether gender bias continues to be an issue in professions where women’s representation has now substantially increased. Thus, it remains unclear whether addressing the issue of women’s underrepresentation in a profession is a reliable indicator that issues of gender bias and differential treatment are now resolved.

Moreover, and quite critically, we examine whether any persisting gender bias is broadly evident, or whether it is perpetuated by a particular subset of individuals. Specifically, we test whether those who believe that women in their field no longer face bias are, perhaps ironically, the most likely to convey biased perceptions and evaluations. While such a belief may seem reasonable to adopt, especially upon seeing women’s representation in the field grow (a very real and notable stride toward gender equality), it may actually make one more susceptible to conveying bias.

We examine these processes in a profession once heavily male-dominated but now with a substantial representation of women, veterinary medicine. Following a preliminary field survey, we conducted a randomized double-blind experiment using a sample of business owners, employers, and managers in the profession—individuals who are in real positions of power to evaluate and shape the experiences and careers of women and men in their field.

### Is gender bias still a problem after women become well represented?

With an increase in women’s representation, it is possible that issues of gender bias will dissipate. This may occur through change in professional culture, including shifts in the perceptions of women’s abilities [e.g., others may not so readily assume (consciously or otherwise) that women in that field are less capable than men nor struggle to recognize women’s skills and achievements] (*18*). Consistent with this perspective, evidence shows that in professions where women are well represented, there is very little bias in how male versus female employees are evaluated (*4*). However, this evidence comes from professions where women’s representation has been relatively stable over time (e.g., nursing and social work), so it does not evince whether such bias will exist in professions where the gender composition has substantially changed. Nevertheless, it provides some indication that when women’s representation in a field is relatively high, gender bias may not be an issue.

On the other hand, it is possible that despite women becoming well represented, gender biases will persist. This may be because there are commonly held assumptions in many cultures that men are more capable than women (*19*), which can give way to biases that disadvantage women [or advantage men (*20*)] (*21*). Importantly, everyone is susceptible to internalizing these stereotypical perceptions, including women and men and those who reject overtly sexist attitudes (*19*, *22*, *23*). Moreover, regarding perceptions of women in scientific fields, evidence shows that even when the proportion of women working in those fields is relatively high, gender stereotypes that favor men (as being more suitable or fitting to the field) can persist (*24*, *25*). Thus, given that certain gender stereotypes may remain unchanged by the proportion of women working in that field, overt expressions of gender bias and discrimination might also persist. Consistent with this perspective, albeit from an educational context rather than the workplace [see also (*26*)], there is evidence of bias in competence evaluations of female (versus male) undergraduates in biology (*27*). Given that women now earn undergraduate degrees in biology at rates equal to or greater than men (*28*, *29*), this suggests that biased evaluations of women can persist even when women’s representation in that context has grown.

### A paradox: Those who think bias is no longer a problem may be most likely to express it

Once women become well represented in a profession, gender bias may also remain evident because that very shift in gender composition, that growth in the number of women, may lead people to more readily infer (perhaps erroneously) that discrimination is now a thing of the past [for a related perspective, see (*30*)]. This idea aligns with previous work suggesting that individuals who hold this type of belief—that women no longer face discrimination in society more generally—tend to lack awareness of the ways in which discrimination toward women can manifest contemporarily, and often subtly (*31*, *32*). It follows that if individuals are unaware of the subtle manifestations of gender bias, they would also be less likely to recognize circumstances in which biases might be guiding their own perceptions and evaluations of an individual. In this way, if individuals infer that a robust representation of women means that gender bias is no longer an issue in their profession, they may inadvertently increase their susceptibility of expressing gender bias—a seeming paradox that arises from perceiving progress on gender equality within one’s profession, or, more precisely, one that arises from misperceiving the true level of progress that has been reached on gender equality (i.e., overestimating the progress that has been made).

This process may be particularly evident in fields of science and medicine, where objectivity is highly valued and routinely practiced as the basis for making observations and evaluations. This is because individuals who feel confident in their capacity to be objective can be especially prone to expressing bias (*33*, *34*). Thus, this process—whereby gender bias is driven by those who think bias is no longer an issue—may be particularly evident in professions where objectivity is routinely practiced and thus readily assumed to underlie one’s perceptions and evaluations. Overall, this perspective aligns with research demonstrating that academics in science disciplines who think discrimination against women is no longer an issue in society are especially prone to displaying gender bias—evaluating a female undergraduate as less competent than an equally qualified male student (*5*). This similarly aligns with research showing that when scientific evaluation committees believe discrimination against women in science is no longer an issue (and hold implicit gender biases), they demonstrate greater gender bias—promoting fewer women to elite research positions (*35*).

The current research builds on this previous body of work in several innovative ways. In part, it examines a profession where women are now well represented rather than a mixture of fields where gender representation varies (e.g., physics and biology). Therefore, it is poised to determine whether increasing women’s representation in a field does represent a robust strategy for eliminating gender bias. It also more precisely examines individuals’ beliefs about whether gender bias is an issue within their own field [rather than in society more generally or across an array of scientific disciplines (focused on those where women remain underrepresented)].

To find evidence of gender bias under these conditions would reveal several unique insights. First, it would show that establishing a strong representation of women does not equate to resolving issues of gender inequality in a profession. This would seem particularly important to consider, given that concerted efforts are underway in a number of fields to increase women’s representation (*14*, *36*). Second, quite critically, it would demonstrate that believing gender equality has been achieved within one’s own field may be a key risk factor for expressing gender bias—a risk factor that can be easily measured and can be readily acknowledged and discussed with those who hold such a belief. Therein, as a practical implication, such insights could aid in the development of targeted bias interventions designed to maximize effectiveness among those who are most likely to demonstrate gender bias.

### The current studies

In the current studies, we use a preliminary field survey (study 1) and preregistered randomized double-blind experiment (study 2) to test whether gender biases are evident in a profession once male-dominated but now with a substantial representation of women. We also test whether those who believe discrimination against women in their field is no longer an issue are the most likely to express gender bias (study 2). We test these questions using samples of women and men working in the field of veterinary medicine (U.K.-based). In 1960, only 5% of U.K. vets were women; by 2017, it was more than 50% (paralleling trends in the United States) (*2*, *37*, *38*). This professional context offers a rather conservative test of whether gender biases will still be evident. This is, in part, because women have been well represented among vets for some time (representing 50%+ of vets for more than a decade) (*39*), and so, in this time, more traditional, biased perceptions and assumptions of women’s abilities in the field (e.g., lacking competence) may have faded. Thus, given this passage of time, it would seem particularly unlikely that gender biases would be evident, compared to fields where women have only more recently reached greater representation. Moreover, in fields where the gender composition is only now shifting, individuals may feel more imminent threat and thus express more overt hostility or negative reactance toward women (*40*). Yet, in veterinary medicine, this type of perceived threat would seem less likely given that women’s sizable representation has been established for more than a decade. This enables a relatively clean test of extant subtle biases, distinct from discriminatory evaluations rooted in overt hostility (see also the “Study 2 supplemental analyses” section in the Supplementary Materials).

#### *Study 1*

In study 1, we tested for preliminary evidence of extant biases toward women in the profession. We analyzed data from a field survey of professionals in veterinary medicine (*N* = 1147; 66% female). Individuals were asked (i) how often they experience gender discrimination at work (e.g., are treated according to stereotypes) and (ii) the extent to which they feel their overall competence and value is recognized by colleagues (e.g., being admired and highly regarded among colleagues). We predicted that women would experience greater discrimination and less value/admiration among colleagues compared to men statistically matched on various characteristics.

#### *Study 2*

Given that study 1 relied on self-reported experiences, in study 2, we aimed to provide confirmatory evidence by using a controlled experimental design. To test whether male and female vets would be evaluated differently based solely on their gender, we showed managers in the profession a performance review of a vet, randomly assigned a male or female name (“Mark” or “Elizabeth”). Everything about this vet—qualifications, experience, past performance, and merits—were identical, aside from the vet’s purported gender. The review described a vet whose performance reflected a mix of positive qualities and drawbacks, thereby creating some ambiguity about the vet’s overall competence [consistent with previous research (*5*); see also (*41*)]. To ensure the performance review was realistic, it was developed collaboratively with the British Veterinary Association (BVA).

To assess whether any potential bias would be driven by those who think bias is not an issue anymore, managers reported whether they believe women in their field still face bias [endorsement of the statements, “Discrimination against women in the veterinary profession is no longer a problem”; “In this profession, the careers of female vets are still impacted by biases and discrimination toward women” (reverse-scored)]. To minimize potential influence of this measure on evaluations of the target vet, it was administered after managers provided their evaluations. Administering this measure beforehand would have posed a risk to the ecological validity of the results (e.g., by priming managers, in an unrealistic way, to actively consider the possibility of extant gender biases in their profession; this could induce self-monitoring and yield less natural evaluations). Note that managers randomly assigned to the male versus female target conditions did not differ in their endorsement of this belief, *t*_252_ = 0.81, *P* = 0.42 (for more details, see Materials and Methods). This suggests that having managers first evaluate the target did not systematically alter their endorsement of this belief.

To maximize external validity, we recruited managers, employers, business owners, and others in the profession with managerial experience (*N* = 254, 122/132 assigned to male/female target conditions), 92% of whom were actively involved in conducting or overseeing performance reviews. This sample of (volunteer) managers is valuable for several reasons. First, these individuals are in real positions of power, making evaluations of others in their field. This yields high external validity. Therefore, findings provide meaningful, real-world implications. Second, individuals who have actual experience with workplace evaluations tend to show less bias compared to more convenient samples (e.g., undergraduate students) (*4*), which means that this sample provides a particularly conservative test of predictions. Third, this sample provides insight into the range of real-world beliefs that managers have about whether women in their field still face bias, thus providing real insight into the scope of the potential issue (i.e., the proportion of managers who hold beliefs that put them at higher risk for exhibiting gender bias).

To help maximize interest and engagement in the study, managers were contacted directly by the BVA. They were told that the BVA was a collaborative partner and that the “survey” aimed to understand their experiences with “managing others in the veterinary profession...[and] to gain insights about how managers...work with other vets to develop successful and thriving practices” (for more details, see the “Study 2 data collection procedures and participant information” section in the Supplementary Materials). While it was imperative to provide managers with a fictitious performance review, so to isolate employee gender as the only experimental factor, managers were told that the performance review was real, was recently completed, and was provided by a BVA-affiliated clinic (upon completion of the study, managers were fully debriefed). Thus, we took several steps to ensure that the study and its stimuli were realistic and engaging. For more details on the applicability of these experimental data to current issues in the profession (e.g., an extant pay gap), see Discussion.

**Fig. 1:**
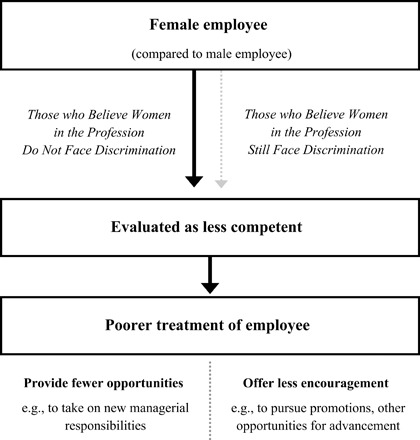
Conceptual model illustrating that in professions once male-dominated, now with a strong representation of women, gender-biased evaluations of an employee’s competence will persist. These biased evaluations will favor a male employee over an otherwise identical female employee and be evident among those in the profession who believe discrimination against women in their profession is no longer an issue. Biased evaluations will subsequently translate into biased treatment of the employee.

Figure 1 provides a conceptual overview of predictions. Overall, we expect that gender-biased evaluations will remain evident in fields once male-dominated but now with a substantial representation of women, including veterinary medicine. Critically, however, this bias will be evident primarily among those who believe women in their field do not experience bias anymore (such that a male employee will be evaluated as having greater competence than an identical female employee). In addition, we expect that gender-biased evaluations will be reflected in differential treatment of the employee, specifically the types of treatment that are grounded in perceptions of an employee’s competence. This more distinctive treatment includes providing opportunities to take on unique supervisory responsibilities (if s/he was under one’s management) and other tasks that exact a relatively distinct level of ability or competence.

## RESULTS

### Study 1: Preliminary self-reported evidence of gender bias

We analyzed study 1 data using a multivariate analysis of covariance, comparing the experiences of women and men in the profession while controlling for relevant factors (e.g., role in the profession and hours worked per week). Results demonstrated that women’s experiences differed from those of their male counterparts overall, *F*_2,1141_ = 29.25, *P* < 0.001, *d* = 0.45. Specifically, women (*M* = 2.08, SD = 1.09) were more likely than men (*M* = 1.58, SD = 0.78) to experience discrimination, *F*_1,1142_ = 54.83, *P* < 0.001, *d* = 0.44. Women (*M* = 4.50, SD = 1.22) were also less likely than men (*M* = 5.03, SD = 1.12) to experience recognition among colleagues for their value and worth, *F*_1,1142_ = 8.25, *P* = 0.004, *d* = 0.17. Thus, results provided initial evidence that despite notable gains in women’s representation in this field, experiences of gender bias may persist.

Study 1 provided direct insight into how women’s experiences working in this field differ from those of their male counterparts. However, the self-reported nature of these data made it vital to test for corroborating experimental evidence—specifically testing whether gender bias remained evident when examining others’ evaluations of an individual (versus an individual’s self-reports) and when comparing evaluations of two individuals who are truly identical in every way, aside from their gender. Study 2 did exactly that in a randomized double-blind experiment.

### Study 2: Corroborating experimental evidence of gender bias

We analyzed study 2 data using PROCESS (*42*), controlling for managers’ differing characteristics. Experimental condition (target gender; *X*) was coded 0 (female/“Elizabeth”) and 1 (male/“Mark”), and managers’ beliefs about ongoing gender discrimination in their field (M) were examined at ±1 SD (mean-centered; so to examine θ_*X*➔*Y*_ | M). The distribution of these beliefs yielded values at ±1 SD of approximately 2.55/5.59 (*M* = 4.07; 1 to 7 scale; for descriptive clarity, values here are not mean-centered). This corresponded to a general rejection versus endorsement of the idea that women in their profession no longer face discrimination. Thus, these values are meaningful not only because they reflect true values in the population but also because they represent categorically distinct beliefs about the existence of gender discrimination in the field.

#### *Prevalence of beliefs about gender bias in the profession*

Initial descriptive analyses revealed that a plurality of managers believed that gender discrimination was no longer an issue in their profession [scoring above the scale’s midpoint (neither agree nor disagree)]. Specifically, 44.5% of managers believed this, of whom 61.1% were men. Another 40.6% of managers rejected this belief (scoring below the midpoint), of whom 23.3% were men. Another 15.0% were neutral/uncertain (scoring at the midpoint), of whom 42.1% were men. Further analyses showed that while both men and women endorsed this belief (and rejected it), men were significantly more likely to endorse it (and women more likely to reject it), χ^2^ (1) = 31.34, *P* < 0.001. Similarly, examining endorsement on a continuum (versus categorically) showed that men’s endorsement of this belief (*M* = 4.75, SD = 1.33) was significantly greater than women’s (*M* = 3.56, SD = 1.45), *t*_252_ = 6.72, *P* < 0.001, *d* = 0.85.

Note that in analyses testing whether male versus female managers differed in their tendency to show biased evaluations of the target vet (testing managers’ gender as a moderator; analyses otherwise paralleled primary analyses described below), we found no evidence of differences between male and female managers (for more details, see the “Study 2 supplemental analyses” section in the Supplementary Materials). Rather, as described below, managers’ biased evaluations of the target vet were squarely rooted in their belief that women in the profession no longer face discrimination.

#### *Evaluations of competence*

Paralleling previous work (*5*), the performance review was designed to create ambiguity about the target employee’s competence, and so, primary analyses tested whether managers’ competence evaluations differed as a function of the target’s purported gender and whether such a difference was evident squarely among those who believe gender bias is no longer an issue in the profession. Predictions were tested in PROCESS model 1 with 5000 resamples (95% confidence intervals in brackets).

**Fig. 2:**
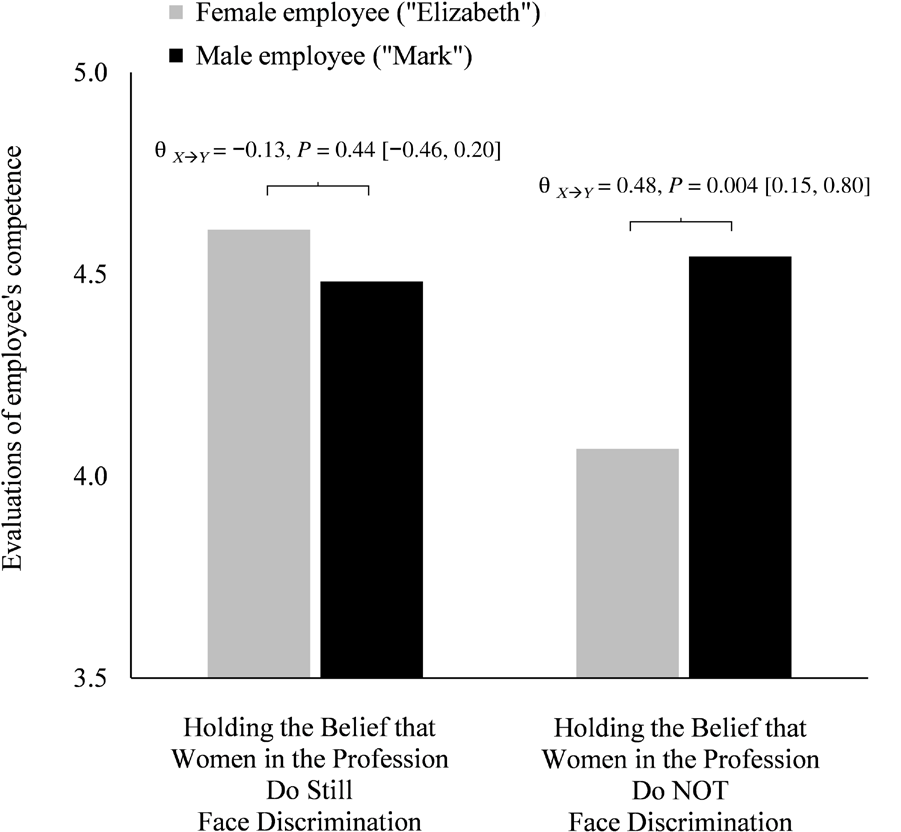
Evaluations of employee competence (general measure) by the purported gender of the target employee and managers’ beliefs about whether women in their profession still face discrimination. Scale is 1 to 7 (*n* = 236); higher values indicate higher competence evaluations. Among managers who believe discrimination against women is no longer an issue, the male employee was evaluated as more competent than the otherwise identical female employee. Analyses probed the interaction (by managers’ beliefs) at ±1 SD. These values correspond to a general endorsement/rejection of these beliefs. For ease of interpretation and because the values represent categorically distinct beliefs, they are presented as bars (estimated means at ±1 SD with covariates at their sample means). The differences in means correspond to the following values: “Holding the Belief that Women in the Profession Do Still Face Discrimination,” θ_*X*➔*Y*_ = −0.13, SE = 0.17, *P* = 0.44 [−0.46, 0.20]; “Holding the Belief that Women in the Profession Do NOT Face Discrimination,” θ_*X*➔*Y*_ = 0.48, SE = 0.17, *P* = 0.004 [0.15, 0.80]. For an analogous depiction with the above confidence intervals (around the conditional effect of target gender, θ_*X*➔*Y*_ | M), see fig. S2.

Analyses of the first competence indicator (overall competence) evinced differences in the perceived competence of the male versus female employee but only among those who believed gender bias was no longer an issue: condition*bias-belief, *B* = 0.20 [0.05, 0.35], SE = 0.08, *P* = 0.01, Δ*R*^2^ = 0.03 (*F*_1,227_ = 6.58), *f* ^2^ = 0.03 (main effects: condition, *B* = 0.17 [−0.06, 0.41], SE = 0.12, *P* = 0.14; bias-belief, *B* = −0.08 [−0.16, 0.00], SE = 0.04, *P* = 0.06). Managers who rejected this belief did not differ in their competence evaluations of the male and female target employee (θ_*X*➔*Y*_ = −0.13 [−0.46, 0.20], SE = 0.17, *P* = 0.44). By comparison, managers who endorsed it—those who believed women in their profession no longer experience bias—demonstrated a systematic bias, evaluating the male employee as significantly more competent than the otherwise identical female employee (θ_*X*➔*Y*_ = 0.48 [0.15, 0.80], SE = 0.17, *P* = 0.004; Fig. 2).

As another indicator of the perceived competence and worth of this employee, managers indicated the extent to which they anticipated this employee was valued, admired, and looked up to among colleagues (paralleling the measure of perceived value/worth among colleagues from study 1). Mirroring the effect described above, results evinced differences in how the male versus female employee was evaluated, specifically among managers who believed gender bias was no longer an issue: condition*bias-belief, *B* = 0.27 [0.11, 0.44], SE = 0.08, *P* = 0.001, Δ*R*^2^ = 0.04 (*F*_1,236_ = 10.84), *f* ^2^ = 0.05 (main effects: condition, *B* = 0.09 [−0.16, 0.34], SE = 0.13, *P* = 0.47; bias-belief, *B* = 0.02 [−0.07, 0.11], SE = 0.05, *P* = 0.62). Again, while those who rejected this belief did not differ in their evaluations of the male and female target (θ_*X*➔*Y*_ = −0.33 [−0.68, 0.03], SE = 0.18, *P* = 0.07), managers who believed gender bias is no longer a problem evaluated the male employee as having greater value and worth than the otherwise identical female employee (θ_*X*➔*Y*_ = 0.51 [0.16, 0.86], SE = 0.18, *P* = 0.005).

**Fig. 3:**
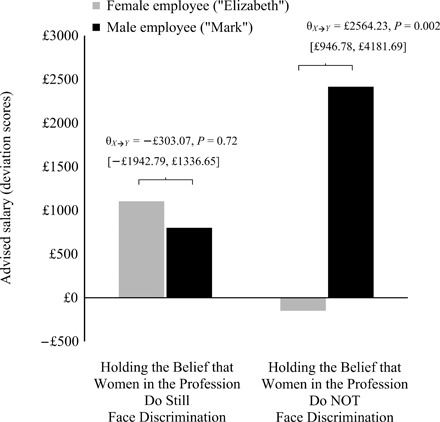
Managers’ advised salary for the target employee, by the purported gender of the employee and managers’ beliefs about whether women in their profession still face discrimination (*n* = 229). To account for individual differences in base salary rates, managers reported the typical salary in their practice for employees with similar experience as the target, and this was subtracted from the advised salary; thus, any differences in base salary rates (can be substantial across different regions of the country) were accounted for by analyzing respondent-specific deviations in advised salary. *Y*-axis values therefore represent deviations in advised salary (from individually adjusted base salary). A value of £0 indicates that managers advised paying the target employee the same as others in their practice with comparable experience. Among managers who believe discrimination against women is no longer an issue, they advised that the male employee receive a higher salary than the otherwise identical female employee. Analyses probed the interaction (by managers’ beliefs) at ±1 SD. These values correspond to a general endorsement/rejection of these beliefs. For ease of interpretation and because the values represent categorically distinct beliefs, they are presented as bars (estimated means at ±1 SD with covariates at their sample means). The differences in means correspond to the following values: “Holding the Belief that Women in the Profession Do Still Face Discrimination,” θ_*X*➔*Y*_ = −£303.07, SE = £832.00, *P* = 0.72 [−£1942.79, £1336.65]; “Holding the Belief that Women in the Profession Do NOT Face Discrimination,” θ_*X*➔*Y*_ = £2564.23, SE = £820.71, *P* = 0.002 [£946.78, £4181.69]. For an analogous depiction with the above confidence intervals (around the conditional effect of target gender, θ_*X*➔*Y*_ | M), see fig. S3.

As a monetary indicator of perceived competence and worth (paralleling previous work) (*5*), managers indicated the salary they would advise for this employee if s/he was in their own practice. Managers also reported the typical salary for employees in their practice with similar levels of experience as the target, and this was subtracted from the advised salary. Thus, analyses accounted for differences in base salary rates by examining respondent-specific deviations in advised salary (the same pattern of results emerged when analyzing raw advised salaries with typical salary used as a covariate; see the “Study 2 supplemental analyses” section in the Supplementary Materials). Mirroring the effects described above, results evinced bias in advised salaries, specifically among managers who believed gender bias was no longer an issue: condition*bias-belief, *B* = £934.98 [£183.01, £1686.95], SE = £381.55, *P* = 0.02, Δ*R*^2^ = 0.03 (*F*_1,220_ = 6.00), *f* ^2^ = 0.03 (main effects: condition, *B* = £1130.58 [−£19.61, £2280.77], SE = £583.61, *P* = 0.05; bias-belief, *B* = £56.89 [−£357.81, £471.58], SE = £210.42, *P* = 0.79). Thus, while those who rejected this belief did not differ in advised salaries (θ_*X*➔*Y*_ = −£303.07 [−£1942.79, £1336.65], SE = £832.00, *P* = 0.72), managers who endorsed it advised paying the male employee ~£2564 or $3475 more than the otherwise identical female employee (θ_*X*➔*Y*_ = £2564.23 [£946.78, £4181.69], SE = £820.71, *P* = 0.002; Fig. 3). This equated to a gender pay gap of approximately 8% or, more formally, unequal pay of 8% (for equally qualified workers). As a more direct translation, this equated to paying the male employee ~$1.75 more than the female employee every hour for the next 2000 consecutive hours or one full year of work. A second measure of perceived financial worth (willingness to offer the employee a raise) showed the same pattern of results: condition*bias-belief, *B* = 0.35 [0.07, 0.63], SE = 0.14, *P* = 0.01, Δ*R*^2^ = 0.02 (*F*_1,235_ = 6.02), *f* ^2^ = 0.02 (main effects: condition, *B* = −0.02 [−0.45, 0.41], SE = 0.22, *P* = 0.93; bias-belief, *B* = −0.12 [−0.27, 0.04], SE = 0.08, *P* = 0.13), although the difference in offered pay raise by target gender was not significant among those who rejected (θ_*X*➔*Y*_ = −0.55 [−1.16, 0.06], SE = 0.31, *P* = 0.08) or endorsed (θ_*X*➔*Y*_ = 0.52 [−0.09, 1.12], SE = 0.31, *P* = 0.09) beliefs about women still facing bias in the field.

Last, to produce a more robust indicator of competence and worth, as in previous work (*5*), the four aforementioned competence indicators were standardized and averaged to form a composite. Consistent with results for each individual measure, this composite measure evinced differences in competence evaluations among those who believed gender discrimination was no longer an issue: condition*bias-belief, *B* = 0.22 [0.11, 0.33], SE = 0.06, *P* < 0.001, Δ*R*^2^ = 0.06 (*F*_1,213_ = 15.10), *f* ^2^ = 0.07 (main effects: condition, *B* = 0.12 [−0.05, 0.30], SE = 0.09, *P* = 0.16; bias-belief, *B* = −0.04 [−0.10, 0.03], SE = 0.03, *P* = 0.27). Again, while those who rejected this belief did not differ in their evaluations of the male and female employee (θ_*X*➔*Y*_ = −0.22 [−0.47, 0.03], SE = 0.13, *P* = 0.08), managers who endorsed it evaluated the male employee as more competent than the otherwise identical female employee (θ_*X*➔*Y*_ = 0.47 [0.22, 0.71], SE = 0.12, *P* < 0.001).

#### *Competence evaluations predict treatment of the employee*

With evidence that managers’ own beliefs about extant gender biases undergird their likelihood of expressing gender-biased evaluations, further analyses tested whether managers’ biased competence evaluations translated into biased treatment of the employee (if s/he was in their own practice; e.g., whether they would let her/him take on more supervisory responsibilities). Specifically, moderated mediation (model 7, using competence composite measure) tested for an indirect effect of employee gender on managers’ intended treatment of the employee, via perceived competence—an effect expected to be evident among those who thought gender discrimination was no longer an issue in their field.

**Fig. 4:**
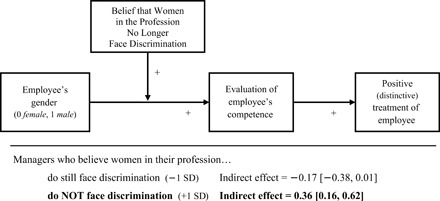
Managers’ differential treatment of the target employee, as a function of the employee’s purported gender and managers’ (biased) evaluations of the employee’s competence (rooted in their belief that women in the profession no longer face discrimination) (*n* = 222). Managers’ competence evaluations were key to predicting how they would treat the employee {e.g., willingness to let her/him take on more supervisory responsibilities and be more involved in managing the business/financial side of the practice (if s/he was in their practice); *B* = 0.77 [0.56, 0.98], SE = 0.11, *P* < 0.001}. However, these competence evaluations were themselves systematically biased among those who thought gender bias was no longer an issue (condition*bias-belief, *B* = 0.22 [0.11, 0.33], SE = 0.06, *P* < 0.001), which translated into differential, discriminatory treatment. In other words, there was a significant indirect effect of target gender on treatment but only among those who believed that gender bias was no longer an issue: indirect effect = 0.36 [0.16, 0.62]. Among managers who rejected this belief, the employee’s gender had no bearing on how s/he would be treated (indirect effect = −0.17 [−0.38, 0.01]).

Results demonstrated just that. While managers’ competence evaluations were critical to predicting how they would treat the employee overall (*B* = 0.77 [0.56, 0.98], SE = 0.11, *P* < 0.001), these competence evaluations were themselves systematically biased among those who thought gender bias was no longer an issue (condition*bias-belief, *B* = 0.22 [0.11, 0.33], SE = 0.06, *P* < 0.001), which translated into biased treatment. In other words, there was a significant indirect effect of target gender on treatment (direct effect: *B* = −0.17 [−0.45, 0.11], SE = 0.14, *P* = 0.24) but only among those who believed gender bias was no longer an issue: indirect effect = 0.36 [0.16, 0.62]. Among those who rejected this belief, the employee’s gender had no significant bearing on how s/he would be treated (indirect effect = −0.17 [−0.38, 0.01]; Fig. 4).

As a second indicator of how managers would treat this employee, they were asked to indicate what advice they would give if the employee expressed interest in pursuing a key promotion in the near future. Specifically, they were asked how readily they would encourage her/him to seek this promotion (to the position of principal vet; response options ranged from advising s/he pursue the position within the next year, to advising that s/he would not be ready to take on this position anytime in the next 6 years). Results mirrored those described above. The employee’s gender had a significant indirect effect (direct effect: *B* = −0.20 [−0.49, 0.08], SE= 0.14, *P* = 0.16) on the advice managers would give, favoring the male employee, but only among those who believed gender bias was no longer an issue: indirect effect = 0.27 [0.10, 0.49]. Among managers who rejected this belief, the employee’s gender had no significant bearing on the advice s/he would be given (indirect effect = −0.13 [−0.30, 0.00]).

## DISCUSSION

The current studies provide evidence that gender biases can persist even in a field where women have made substantial gains in their representation. This evidence comes from ecologically valid field survey data combined with controlled experimental data, which also uses ecologically valid respondents—managers and others who are in actual positions of power to evaluate and shape the careers of women and men in their field. Moreover, and quite critically, this research demonstrates that managers who think bias is no longer an issue in their profession are, perhaps ironically, the key drivers of bias.

Together, our research provides several unique insights. In part, it demonstrates that when women’s representation in a field substantially increases, it cannot be taken to indicate that issues of gender bias have been resolved. This may be particularly important to consider as we see concerted efforts underway in a number of STEMM fields to increase women’s representation (*14*, *36*). While gender biases may be even more pervasive when women are highly underrepresented, and so increasing women’s representation in these fields may be beneficial in some respects, the current studies indicate that making progress on “the numbers” should not be considered a robust or adequate solution to issues of gender inequality. In fact, the current evidence indicates that when a field (or particular organization in it) makes gains in women’s representation, they may need to take additional precautions to ensure that this does not get interpreted to mean that gender bias is no longer a problem. While this may seem like a reasonable inference to make, our results show that to make such an inference actually puts an individual at higher risk for demonstrating gender bias.

Following from this, the current research illustrates that an individual’s beliefs about gender equality in their field may be a notable, and readily measurable, risk factor. Those who believe gender bias is no longer an issue in their profession, or who generally underestimate its pervasiveness, may be at highest risk for exhibiting such bias. This is a key insight, with practical implications. For example, this may be important for understanding and precisely identifying who in the profession is perpetuating the ongoing gender pay gap (*43*). It is also notable that the actual pay gap in veterinary medicine (approximately 8% for junior full-time vets) closely mirrors the one found in the current research. This high degree of consistency, between the magnitude of the real-world issue and the current findings, also suggests that while managers in this study were not evaluating a real employee (with real implications for themselves or the employee), their evaluations may nevertheless mirror real-world evaluations and treatment of their employees [e.g., managers’ actual perceptions of employees’ competence and the salaries they advise for (prospective) employees]. The gender-biased evaluations shown in the current experiment also generally map onto women’s lived experiences of discrimination in the profession, as evinced in study 1. Thus, together, this suggests that both the results and particular insights of the current research—including that those who believe gender bias is no longer an issue in their profession are at highest risk for perpetuating it—will be vital to understanding, and ultimately addressing, extant issues of gender bias in the profession.

There are both men and women who believe that discrimination against women is no longer an issue in their profession (in the current research, 45% of managers held this belief, 66% of whom were men), and it is the belief itself, not one’s gender, that predicts who is most likely to demonstrate gender bias (see also the “Study 2 supplemental analyses” section in the Supplementary Materials). Although it will be important to further probe the nature of this gender bias effect in future research {e.g., for whom it is explained by a genuine naiveté of extant forms of gender discrimination versus a more explicit motivation to deny that gender discrimination still exists [in line with ideas put forth in literature on system justification (*44*, *45*) and modern sexism (*31*, *32*)]}, it is critically informative that simply holding this belief that gender discrimination is no longer an issue in one’s own field reveals a pattern of robustly biased evaluations of women. When considering how to develop targeted gender bias interventions, it seems particularly useful to have identified this risk factor—a belief that is explicit, is easily measured, is profession specific (and thus likely to be relevant to individuals), and can be readily acknowledged and discussed with those who hold it.

In addition, the current research shows that biased competence evaluations can translate into differential treatment of an employee. It is also informative that managers in the current research did not directly indicate differential treatment of an employee based on gender but instead indicated that they would treat an employee based on his or her perceived competence. Critically, however, these competence evaluations were themselves systematically biased (among those who thought gender bias was no longer an issue). This suggests that managers may overtly value the notion that employees should be treated based on their competencies and merits. However, despite seeming like a fair standard to maintain, it can be an insidious one. This is because the very foundation of that standard—perceptions of an employee’s competence—can be fundamentally biased.

Last, it is important to note that while there is a considerable amount of research, and debate, around whether gender bias plays a role in explaining women’s underrepresentation in certain fields (*46*), the current research focally speaks to a different question. Rather than focusing on the antecedents of women’s representation in a profession, this research examines whether gender bias plays a role even after issues of women’s representation have largely been resolved. In this way, it helps address a distinct and more forward-looking question: When a traditionally male-dominated field ultimately establishes a strong representation of women, will those women—having already surpassed any potential barriers to entering the field—finally be on an equal footing with their male colleagues? Will they have the same opportunities to thrive and face the same challenges to advancement? Overall, the current research indicates that this is not the case. Even when well represented, women can continue to face unique challenges in how they are perceived, evaluated, and treated because of their gender.

Going forward, it will be important to examine how and why individuals come to believe that gender bias is no longer an issue in their field. While the current studies demonstrate clear consequences of holding this belief, it does not examine its antecedents. Given the context in which these consequences are demonstrated—a field where women’s representation has grown—and given that several professions are now making efforts to increase women’s representation, it may be particularly valuable to assess whether individuals seeing the number of women in their field grow (i.e., subjectively perceiving growth) is partly what gives rise to this belief. Finding that this belief becomes more likely or prominent when women’s representation perceptibly grows would illustrate how gains in women’s representation—a very real and notable stride toward equality—can also give way to an insidious belief that undermines equality. Future study of this and other related processes would ultimately benefit from a mixed methodological approach, including additional experimental work (e.g., manipulating the perceived representation of women in a profession and manipulating individuals’ belief that gender bias is still an issue), and from studying other relevant professions (e.g., biological sciences and medical fields where women’s representation has grown).

Similarly, it will be important to consider for whom an increase in women’s representation yields a belief that gender bias is no longer an issue and, by comparison, for whom this belief will exist irrespective of women’s representation. In line with past theorizing (*32*), some individuals may genuinely, though perhaps naïvely, infer from seeing a growth in women’s representation that gender bias is no longer an issue. Thus, for these “naïve deniers” of extant bias, seeing women’s representation increase would be key to producing the belief. However, these individuals can also have a genuine motivation to promote gender equality (*32*). This is important because it suggests that awareness raising interventions may be effective in changing their beliefs and ultimately the discrimination that, as shown, can accompany this belief (i.e., for naïve deniers, effective interventions may include increasing awareness of extant forms of gender bias, and awareness that thinking bias does not exist makes them more likely to express it). By comparison, other individuals may not be naïve deniers so much as “motivated deniers” of extant bias. Such individuals may strategically use information, including about women’s representation but also other selective information or ideas, to justify what is more fundamentally a sexist, anti-egalitarian attitude [in line with theorizing around modern sexism (*31*, *32*) and system justification] (*44*). For motivated deniers of extant bias, women’s representation in a field is less central to determining whether they hold the belief (though a growth in women’s representation could certainly strengthen it) because, even in the absence of women being well represented, motivated deniers will perform the mental gymnastics necessary to sustain the belief [using requisite rationalizations; e.g., cognitively emphasizing that gender (or sex) discrimination is, by law, illegal and so conclude that it is unlikely to be happening in the workplace]. This ultimately suggests that for those who are motivated, whether consciously or not, to deny that gender discrimination is still an issue in their profession, awareness raising interventions may be relatively ineffective. For motivated deniers, other interventions may be necessary to mitigate their potential expressions of bias (e.g., implementing systems and protocols that minimize space for subjectivity in employee evaluations).

Together, the current research illustrates that even when issues of women’s representation in a field have largely been resolved—even when there is a wealth of women who have made it into the field’s “pipeline,” with careers fully underway—gender biases can thrive. Yet, this research also provides nuance to that point. Yes, it appears that gender bias is still a problem, but not everyone is contributing to it equally. There is instead a focal group of individuals who are perpetuating this bias, and it is perhaps ironically those who think it is not happening. Ultimately, this highlights an insidious paradox that can arise when individuals misperceive the level of progress made on gender equality in their profession such that those who mistakenly think gender bias is no longer an issue become the highest risk for perpetuating it. Thus, as other STEMM professions strive to establish greater representations of women, it will be important that they carefully consider what any change in representation signifies in terms of progress for their field, what it does not signify, and what new barriers to gender equality might surface in its wake.

## MATERIALS AND METHODS

### Study 1 design

Individuals in the field of veterinary medicine completed a semiannual survey organized and distributed by the BVA. The survey was designed for the BVA’s own internal purposes but also included study 1 questions. Individuals were asked how often they experience gender discrimination at work [three items, adapted from (*47*): treated according to stereotypes based on your gender, deprived of opportunities available to others because of your gender, and viewed negatively because of your gender; 1 (Never) to 5 (Very often); α = 0.88] and the extent to which they feel their overall value and worth is recognized by colleagues [four items, adapted from (*48*): extent to which they dis/agree that they are, among colleagues: held in high regard, seen as a role model for others, looked up to, and admired; 1 (Strongly disagree) to 7 (Strongly agree) scale; α = 0.93].

Study 1 aimed to examine the experiences of individuals currently working in the profession, so individuals not working were omitted. The sample for analyses (*N* = 1170, *n* = 1147 for main analysis, as some did not respond to all questions) was 66.8% female, 87.4% working in clinical practices, and 83.8% working full-time (35+ hours per week; *M*_age_ = 42.57, SD = 11.91). On average, individuals had graduated from veterinary school 16.87 years ago (SD = 12.65). Roles in the sample, following a coding scheme from the BVA, reflected employees (62.4%), managers of other vets (5.5%), and self-employed/business owners/partners (32.1%). All respondents indicated that they work alongside other employees and thus had a basis for answering questions about their experiences among colleagues (responding “yes” to, “In your current role do you work alongside colleagues or other employees?”). While sample size was determined/managed by the BVA, sensitivity analyses indicated the study was powered to detect effects within the range of those found (e.g., *d* ≥ 0.17 in analysis of variance with covariates; α = 0.05, 1 − β = 0.80).

### Study 1 statistical analyses

We compared the experiences of women and men in the profession using a multivariate analysis of covariance (covariates: role/position in the profession, hours worked per week, and years since graduating from vet school). We used an α level of 0.05 (two-tailed) for analyses (no data transformations). Effect sizes computed in SPSS were converted using established equations (*49*).

### Study 2 design

Using a randomized double-blind experimental design, managers and others with managerial experience (e.g., business owners and employers) in veterinary medicine were shown a performance review of a vet—randomly assigned a male or female name (with corresponding male or female pronouns used). The review described a junior vet whose past performance reflected a mix of qualities and drawbacks, thus creating ambiguity about the vet’s overall competence. Figures S1 and S2 show the performance review (male version) and cover story that preceded it. Everything about this vet was identical aside from their gender. Thus, any differences in managers’ evaluations of the vet’s competence could be attributed to the vet’s gender.

After omitting respondents who did not match inclusion criteria [e.g., those without managerial experience, *n* = 12; those who failed manipulation checks (the correct name/gender of the target employee they evaluated; *n* = 15 assigned to the male target condition, *n* = 18 assigned to female target condition)], there were 254 respondents (57.1% female; 89.4% in clinical practice; *M*_age_ = 45.78, SD = 10.93). On average, respondents entered the veterinary profession (graduated from vet school) 23 years ago (SD = 11.21). When asked about years of managerial experience in the profession, 46% reported having more than 10 years of experience. Another 12, 10, 13, 5, and 9% reported having 7 to 10, 5 to 7, 3 to 5, 2 to 3, and 1 to 2 years of managerial experience. The remaining 6% had less than 1 year of experience. Asked about their current involvement conducting and/or overseeing performance reviews, 79% reported being “very” or “quite involved.” Another 13% reported being “somewhat” or “a little involved.” Only 8% reported no current involvement. When comparing managers randomly assigned to the two experimental conditions on these demographic variables, they did not differ in any way (all *P*s > 0.10). See the “Study 2 data collection procedures and participant information” section in the Supplementary Materials for more information on recruitment, power, and methodology.

### Study 2 measures

To reinforce the target employee’s gender, questions about the employee regularly used his/her name and corresponding gender pronouns. Questions are described here using the male version. The female versions were identical except for the name (Elizabeth) and/or gender pronouns used. For more information, see the “Study 2 experimental materials and supplemental measures” section in the Supplementary Materials.

Consistent with previous research (*5*), multiple measures were used to discern managers’ evaluation of the employee’s competence, value, and worth: (i) general [Generally speaking, how competent does Mark seem to be?; 1 (Not at all competent) to 7 (Very competent)], (ii) colleague-based [adapted from (*48*): Within Mark’s practice, among colleagues do you imagine he is: looked up to? admired? held in high regard? seen as a role model for others in the practice?; 1 (No, definitely not) to 7 (Yes, definitely); α = .92], (iii) advised salary [Considering Mark’s past performance, future potential, etc., if he was employed in your practice, what salary do you think would be fitting for him? Suggested salary: £ (open-ended numeric response); managers also reported the typical salary in their practice for employees with similar experience as Mark, and the typical salary was subtracted from the advised salary; thus, any differences in base salary rates (which can be substantial across different regions of the United Kingdom) were accounted for by analyzing respondent-specific deviations in advised salary; typical salary was assessed with: In your practice, what is the typical salary for vets who are relatively new to the profession (e.g., graduated 1 to 2 years ago)? Typical salary: £ (open-ended numeric response)], and (iv) pay raise {Some vets in Mark’s practice, though certainly not all, get a 2% pay rise each year. If you were Mark’s employer and he came to you and asked for a pay rise, based on his performance, would you give him one? If so, what percentage would you give him?; 1 [No pay rise at this time (0%)] to 7 (3.0%+)}. Higher values on each measure indicated greater perceived competence/worth.

In addition to examining each measure independently, as in previous research (*5*), these measures were standardized and averaged to form a more robust composite measure of competence, which was used in subsequent analyses testing whether biased competence evaluations translated into differential treatment (for analyses using the general competence measure alone, see the “Study 2 supplemental analyses” in the Supplementary Materials). Specifically, managers indicated their likelihood of treating the vet (if s/he was in their own practice) in ways that emerge from, and functionally convey, recognition of an individual’s distinct level of value and worth. This included expressions of distinctive treatment [If Mark was employed in your practice, along with several other vets, would you: let him start taking on more supervisory/managerial responsibilities in the practice? encourage Mark to take on tasks/responsibilities typically reserved for vets at a slightly higher grade than his? let Mark represent the practice at outside (industry/professional) events? advise other vets in the practice to look to Mark as a valuable source of knowledge and guidance? let him serve as a PDP mentor for more junior colleagues (i.e., RCVS Professional Development Phase mentor)? give him the opportunity to become more involved in managing the business/financial side of the practice?; 1 (No, definitely not) to 7 (Yes, definitely); α = .78] and encouragement to pursue a valuable promotion in the near future {In the next few months, if Mark expressed interest in becoming a principal, when would you advise that he seek such a promotion? In other words, how soon do you think Mark could be ready to take on this type of position?; 1 (I think he could be ready to take on a principal position within the next year) to 6 [I do not think he would be ready to take on a principal position anytime in the foreseeable future (anytime in the next 6 years)], reverse-scored}. Higher values on each measure indicated greater willingness to treat the vet in distinctly positive ways.

Managers also indicated their endorsement of the belief that discrimination against women in the profession is no longer an issue [adapted from (*31*): “Discrimination against women in the veterinary profession is no longer a problem.” “In this profession, the careers of female vets are still impacted by biases and discrimination toward women” (reverse-scored); 1 (Strongly disagree) to 7 (Strongly agree); *r* = 0.63, α = 0.77]. Higher scores indicated a stronger belief that discrimination against women is no longer an issue. Managers randomly assigned to the male (*M* = 4.15, SD = 1.58) versus female (*M* = 3.99, SD = 1.46) target condition did not differ in their endorsement of this belief, *t*_252_ = 0.81, *P* = 0.42.

### Study 2 statistical analyses

We used an α level of 0.05 (two-tailed) for all statistical tests (no data transformations; aside from mean centering). For preliminary analyses (e.g., comparing managers by condition on demographic variables), we used independent-samples *t* tests and chi-square tests as required. Results expressed for descriptive purposes in United States Dollar (USD) were based on the British Pound Sterling (GBP) conversion rate on 13 May 2018 (1.355), the median date of data collection within the sample. Results expressed in terms of a pay gap (also for descriptive purposes) were calculated following guidelines parallel to those for official reporting of gender pay gaps in the United Kingdom (*50*) (mean gender difference in pay [mean advised salary for male target − female target]/mean pay for men [advised salary for male target] ×100). For primary analyses, we used PROCESS (*42*) in SPSS to test moderation (model 1) and moderated mediation (model 7), bootstrapped with mean centering (covariates included managers’ age, gender, years of managerial experience, years since graduating from vet school, and current level of involvement in performance reviews; follow-up analyses without covariates evinced virtually identical results). Effect sizes computed in PROCESS were converted using established equations (*49*). For additional information, see the “Study 2 supplemental analyses” section in the Supplementary Materials.

## Supplementary Material

aba7814_SM.pdf

## SUPPLEMENTARY MATERIALS

Supplementary material for this article is available at http://advances.sciencemag.org/cgi/content/full/6/26/eaba7814/DC1
