# Supplementary material for: In some professions, women have become well represented, yet gender bias persists—Perpetuated by those who think it is not happening
Source: Sci Adv. 2020 Jun 26;6(26):eaba7814. doi: 10.1126/sciadv.aba7814 (PMC7319752; doi:10.1126/sciadv.aba7814)
Supplement: aba7814_SM.pdf [file aba7814_SM.pdf]

Science Advances
AAAS

advances.sciencemag.org/cgi/content/full/6/26/eaba7814/DC1

Supplementary Materials for

In some professions, women have become well represented, yet gender bias persists—Perpetuated by those who think it is not happening

C. T. Begeny*, M. K. Ryan, C. A. Moss-Racusin, G. Ravetz

*Corresponding author. Email: c.begeny@exeter.ac.uk

Published 26 June 2020, *Sci. Adv.* **6**, eaba7814 (2020)
DOI: 10.1126/sciadv.aba7814

This PDF file includes:

Study 2 Data Collection Procedures And Participant Information
Study 2 Experimental Materials And Supplemental Measures
Study 2 Supplemental Analyses
Figs. S1 to S5
References

Study 2 Data Collection Procedures and Participant Information

Data were collected from April to June, 2018. Prospective respondents, those with experience managing veterinarians (e.g., current or former managers, employers, business owners), were contacted via email through member list-servs maintained by the British Veterinary Association (BVA). The BVA sent these recruitment emails, with guidance from the authors on how to advertise them (e.g., providing general information about the study, but without indicating that gender-related processes were being examined, nor that there were multiple versions [experimental conditions] within the study). Via email and at the beginning of the online study, prospective respondents were told that the study aimed to understand their "experiences with managing others in the veterinary profession...[and] to gain insights about how managers, business owners, employers, partners, and others in the profession work with other vets to develop successful and thriving practices." They were told that the study was intended for those who had managerial experience in the profession. After being provided an information sheet, respondents indicated consent to participate and proceeded to the online study.

After omitting respondents who did not match inclusion criteria (those who reported no managerial experience; $n = 12$) or who failed manipulation checks (the correct name/gender of the employee they were asked to evaluate; $n = 15$ assigned to the male target condition, $n = 18$ assigned to female target condition) there were 254 respondents. Some did not answer all questions, so the number of respondents in each analysis varied slightly (lowest $n = 222$). In recruiting this real-world sample – individuals who are in real positions of power to evaluate and make decisions about employees in their profession, thus being a sample with high external validity (compared to more convenient samples; e.g., undergraduate students) – there was some loss in ability to determine final sample size. Nevertheless, sample size exceeded that used in related research,[5] and sensitivity analyses ($\alpha = .05$, $1\text{-}\beta = .80$) indicated the study was adequately powered to detect key hypothesized effects (powered to detect condition*bias-belief interactions with effect sizes of f^2 [for ΔR^2] within the range of those found; e.g., sensitivity analyses for the general measure of competence indicated adequate power to detect effects of $f^2 \geq .03$).

Study 2 Experimental Materials and Supplemental Measures

The performance review (experimental stimuli) was developed collaboratively with the BVA. There were two versions. They differed only in the purported gender of the employee (target vet) under review, indicated by the employee's name (*Mark, Elizabeth*) and corresponding pronouns used. These names were selected in part because they have been used in previous studies[51] and in part because they are common for people born in the UK around the time this employee would have likely been born (mid- to late-1980's, based on the typical age range for graduating from veterinary school and the indicated length of time the employee had been working in the profession since graduation). Both are in the top 25 baby names for the mid 1980's.[52] Figures S1a and S1b show the performance review (male version) and cover story that preceded it, respectively.

Consistent with experimental materials used in similar research,[5,41] and consistent with features common for junior vets, the performance review reflected a mix of qualities and drawbacks, thereby creating ambiguity about the employee's overall competence. To reinforce the target employee's gender throughout the study, questions regularly used his/her name and corresponding pronouns.

Further to those described in the main text, managers / respondents also completed the following measures. Items are described using the male version. The female versions were identical except for the name [Elizabeth] and/or gender pronouns used. A description of corresponding analyses are described in *SM: Study 2 Supplemental Analyses*.

In addition to indicating their willingness to treat the target employee in ways that are more distinguishing (e.g., allowing them to take on unique and valued responsibilities, encouraging them to pursue a valuable promotion; see main text for descriptions of these measures), managers also indicated their likelihood of giving the employee certain forms of support if s/he expressed a need (i.e., because balancing his/her current workload felt challenging). Items began: Altogether, considering Mark's performance along with his concerns about managing workload, if you were his employer what advice might you give him and/or what actions might you take? For instance, would you consider…, e.g., providing him access to more support staff? organising more direct mentorship (from more senior colleagues)? offering Mark more flexible working hours? Items were rated on a 7-

point scale (1 *No, definitely not* – 7 *Yes, definitely*; α = .68). Items were averaged to form a composite. Higher scores indicated a greater willingness to invest in and generally support the employee.

Importantly, compared to the form of treatment examined in primary analyses – that which conveys a distinct level of competence and ability seen in the employee (i.e., it confers value, status and worth upon the employee; e.g., by providing opportunities to take on unique managerial responsibilities) – this form of treatment is not grounded in expressions of, or opportunities to demonstrate, some unique level of competence or ability. Rather, this supportive type of treatment entails an effort to help the employee 'keep up' with colleagues per se. Thus, it conveys a general level of care/concern for the employee – a willingness to 'invest' in the employee by providing resources and accommodation – but it does not convey or demonstrate recognition of a particularly high level of competence or ability seen in the employee.

Two additional items were designed to measure managers' *un*willingness to invest in or support the employee: ...suggesting that Mark shift to part-time work? suggesting that he find a more suitable job elsewhere? As a precautionary step these items were assessed separately because there is some ambiguity about whether they reflect a true unwillingness to support the employee (some may interpret this advice as being 'appropriately supportive' if they see the employee as lacking capacity to manage their current position). Analyses revealed, however, that these items showed the same effect as the others but in the opposite direction, suggesting they were generally interpreted as an expression of 'divestment' or unwillingness to support the employee (see *SM: Study 2 Supplemental Analyses*).

Managers were also asked how important they believed each of the five pieces of information in the employee's performance review were for gauging his/her overall ability and future potential (force-ranked, see Figure S1a for the five pieces of information; 1 *The least important...* - 5 *The most important point to consider when trying to assess Mark's overall capacity / future potential*) and how positively or negatively each of these points reflected on him/her (1 *This point reflects very negatively...* – 7 *This point reflects very positively on this vet*). These were used together to assess whether managers justified their (biased) competence evaluations by strategically shifting the relative weight/emphasis placed on different pieces of information.[53] Specifically, this was assessed by calculating a sum of the evaluations for all five pieces of information with each weighted by its rank

(e.g., a piece of information evaluated as reflecting very positively on the employee (*7*) and ranked as the most important to consider (*5*), *7*5 = 35*). Higher values indicate greater emphasis given to positive information about the employee.

Additionally, managers were asked to indicate their perceptions of the employee's overall career potential (Overall, does it seems like Mark is someone with a lot of potential?, More generally, do you anticipate that Mark will be successful in his career?; 1 *No, definitely not* – 7 *Yes, definitely*; α = .68) and perceptions of their general 'fit' to the profession (Overall, does it seem like Mark...is a good fit for this line of work? belongs in this profession? is someone who will really 'fit in' within the profession?; 1 *No, definitely not* – 7 *Yes, definitely*; α = .92). Items for each measure were averaged to form composites. Higher scores indicate the employee was perceived as having more potential and being a better fit for the profession, respectively.

To more conservatively test whether managers' biased competence evaluations were rooted in their belief that women in the profession no longer face discrimination (effects described in main text) we also assessed their endorsement of more overtly hostile sexist attitudes toward women in their profession (2 items, adapted from the Hostile Sexism subscale:[54] Female vets tend to exaggerate the problems they face in this profession, Female vets tend to request too many accommodations or special favours at work; 1 *Strongly disagree* – 7 *Strongly agree*; α = .72), and their general lack of support for focusing on issues of gender and equality within the profession (first prompted with: Over the past several years there has been more attention on issues of gender and equality in the veterinary profession. This includes differences in pay for male and female vets, and women's preparedness for leadership positions. Please indicate how much you agree or disagree with the following statements on this topic; followed by four items: Overall, the increasing level of attention on issues of gender and equality within the veterinary profession is: unnecessary, excessive / 'too much,' valuable [reverse scored], important [reverse scored]; 1 *Strongly disagree* – 7 *Strongly agree*; α = .90). Items for each measure were averaged to form composites. Higher scores indicated more negative/unsupportive attitudes toward women/gender equality. Notably, managers' belief that discrimination toward women in the profession is no longer an issue was associated with less support for focusing on issues of gender and equality ($r = -.47$, $p < .001$), and greater endorsement of hostile sexism ($r = .39$, $p < .001$). Given these associations, and to more critically assess whether managers' biased competence evaluations

were indeed rooted in their belief that women in their profession no longer face discrimination, supplementary analyses tested whether these effects held true even when controlling for managers' hostile sexist attitudes and general lack of support for gender and equality issues (see *SM: Study 2 Supplemental Analyses*).

To assess managers' perception that an 'ideal vet,' the vet prototype, should possess a variety of stereotypically masculine traits, they were asked to indicate how important they believed it was for a vet to possess certain attributes that are typically associated with men:[55] For a vet to be really successful in this line of work, how important is it that they are...confident, level-headed, independent, decisive, skilled in business matters, stands up under pressure. Items were rated on a 7-point scale (1 *Not at all important* – 7 *Very important*; α = .78). Items were averaged to form a composite. Higher scores indicated stronger endorsement of a stereotypically masculine vet prototype.

Study 2 Supplemental Analyses

Additional Depictions of Primary Analyses

Figures S2 and S3 parallel Figures 2 and 3 in the main text. They show the conditional effects of target gender on competence evaluations by managers' beliefs about whether women in their profession still face discrimination (at +/- 1 *SD*). Rather than illustrating these effects as estimated means (see Fig. 2 and 3), Figures S2 and S3 illustrate them as a function of estimated mean differences ($\theta_{X \rightarrow Y}$ | M).

Figure S4 depicts results akin to those in Figure 2 using a modified analytical approach. Both examine managers' advised salaries for the target employee adjusting for differences in base salary rates (i.e., accounting for managers' reports of the typical salary in their practice for employees with comparable experience to the target), and both show the same significant condition*bias-belief interaction. Results shown in Figure 2 reflect analysis of deviation scores (a manager's advised salary for the target employee, minus their reported typical salary for employees in their practice with similar levels of experience as the target). Results depicted in Figure S4 reflect analysis of raw advised salaries, with managers' reported typical salary entered as a covariate. Mirroring the effects depicted in Figure 2 (main text), results of the latter analysis evinced bias in advised salaries, specifically among managers who believed gender bias was no longer an issue: condition*bias-belief, *B* = £987.77 [£275.30, £1,700.24], *SE* = £361.50, *p* = .01, ΔR^2 = .02 (F(1,219) = 7.47), f^2 = .04 (main effects: condition, *B* = £779.68 [-

£317.98, £1,877.33], *SE* = £556.94, *p* = .16; bias-belief, *B* = £34.07 [-£358.78, £426.91], *SE* = £199.33, *p* = .86). Thus, while those who rejected this belief did not differ in advised salaries ($\theta_{X \rightarrow Y}$ = -£734.91 [-£2,296.72, £826.89], *SE* = £792.45, *p* = .35) managers who endorsed it advised paying the male employee ~£2,300 more than the otherwise identical female employee ($\theta_{X \rightarrow Y}$ = £2,294.26 [£758.89, £3,829.64], *SE* = £779.04, *p* = .004). Figure S5 illustrates this as a function of estimated mean differences ($\theta_{X \rightarrow Y} | M$).

Primary analyses used a standardized composite measure of competence to examine how managers' competence evaluations translated into a willingness to convey distinctive treatment toward the employee (e.g., willingness to let her/him take on more supervisory responsibilities, be more involved in managing the business/financial side of the practice [if s/he was in their practice]). To supplement this, an analogous set of analyses were conducted using the general (face valid) measure of competence alone. Results mirrored those described in the main text using the standardized composite. While managers' competence evaluations were critical to predicting how they would treat the employee overall (*B* = .43 [.28, .59], *SE* = .08, $p < .001$), these competence evaluations were themselves systematically biased among those who thought gender bias was no longer an issue (condition*bias-belief, *B* = .20 [.05, .35], *SE* = .08, *p* = .01), which translated into biased treatment. In other words, there was a significant indirect effect of target gender on treatment (direct effect = -.15 [-.43, .13], *SE* = .14, *p* = .29), but only among those who believed gender bias was no longer an issue: indirect effect = .21 [.05, .41]. Among those who rejected this belief, the employee's gender had no significant bearing on how s/he would be treated (indirect effect = -.06 [-.22, .08]). Regarding the second indicator of how managers would treat the target employee (willingness to encourage her/him to pursue a valuable promotion in the near future), results mirrored those described above. The employee's gender had a significant indirect effect (direct effect = -.15 [-.44, .13], *SE* = .14, *p* = .29) on the advice managers would give, favoring the male employee, but only among those who believed gender bias was no longer an issue: indirect effect = .16 [.05, .34]. Among managers who rejected this belief, the employee's gender had no bearing on the advice s/he would be given (indirect effect = -.04 [-.17, .06]).

Controlling for Hostile Sexism and General Lack of Support for Gender and Equality

To more conservatively test whether biased competence evaluations are robustly rooted in managers' belief that women in the profession no longer face discrimination, we ran analyses that paralleled primary analyses but

also controlled for managers' overtly hostile sexist attitudes toward women in their profession, and their general lack of support for giving attention to issues of gender and equality within the profession. Overall, results showed that even with these covariates, the belief that women no longer face discrimination in the profession remained a significant determinant of gender bias, evincing consistently biased competence evaluations favoring the male employee (all condition*bias-belief terms, B's $\geq .19$, p's $\leq .02$). Consistent with results of primary analyses, among those who endorsed this belief, the following effects were found: General competence, $B = .49$, $p = .003$; Colleague-based competence, $B = .51$, $p = .004$; Advised salary, $B =$ £2,588.20, $p = .002$; Advised raise, $B = .56$, $p = .06$; Standardized composite, $B = .47$, $p < .001$. Altogether, this indicated that the pernicious effects of managers' belief that gender bias is no longer an issue is a robust one, giving way to a consistent pattern of biased evaluations over and above any overtly hostile or unsupportive attitudes that they may have about women in their profession. In future research, it will be valuable to control for (gender-based) system justification beliefs as well.

Testing Alternative Moderators

In addition to managers' beliefs that women no longer face discrimination, two other potential moderators were examined: managers' gender and managers' perceptions of a masculine vet prototype.

Managers' Gender. Consistent with previous work,[5] we found no evidence that fe/male managers differed in their tendency to show biased evaluations. When testing managers' gender as a moderator (analyses otherwise paralleled primary analyses) the following condition*manager-gender interaction terms emerged: General competence, $B = .42$, $p = .08$; Colleague-based competence, $B = .31$, $p = .23$; Advised salary, $B =$ -£36.73, $p = .98$; Advised raise, $B = .42$, $p = .34$; Standardized composite, $B = .28$, $p = .12$. Thus, results demonstrated that managers' gender did not reliably determine who expressed biased competence evaluations. Rather, as described in primary analyses, managers' beliefs that women no longer face discrimination explained these biased competence evaluations.

For descriptive purposes we also examined the gender composition of managers who endorsed this belief (scoring above the midpoint on the scale). Overall, while there were more men than women who endorsed this belief (and more women than men who rejected it; see main text for detail), a sizable proportion of women did hold this belief. This suggests the non-significant effect of managers' gender (as a moderator) was not simply due to

lack of variability among women, or men, in their beliefs that women in the profession no longer face discrimination.

Managers' Perceptions of the Vet Prototype. It has been suggested that pro-male biases can emerge when there is a perception that the 'ideal employee' for a position, the prototype, has a preponderance of stereotypically-masculine traits.[56] This is in part because with a more masculine prototype individuals see male employees as a better fit for that position, and female employees as having a relative lack of fit. As a result of these differing perceptions, gender-biased evaluations of male and female employees can emerge. Therefore, we examined whether gender-biased competence evaluations were more pronounced as a function of managers' tendency to maintain a more stereotypically-masculine vet prototype. When testing managers' vet prototype as a moderator (degree to which it was defined by stereotypically masculine traits), the following condition*prototype interaction terms emerged: General competence, $B = .17$, $p = .80$; Colleague-based competence, $B = -.12$, $p = .51$; Advised salary, $B = -£15.40$, $p = .98$; Advised raise, $B = -.39$, $p = .19$; Standardized composite, $B = -.11$, $p = .39$. Thus, results did not indicate that the stereotypically gendered nature of managers' vet prototype determined their tendency to show biased competence evaluations.

Note that while analyses focused on managers' endorsement of stereotypically masculine traits to the vet prototype, consistent with past work, we also measured items reflecting stereotypically feminine traits (considerate, caring, kind, able to multi-task, good communication skills; $\alpha = .72$). The same pattern of non-significant moderation emerged when analyzing managers' endorsement of stereotypically feminine traits to the vet prototype, and when analyzing the proportional endorsement of stereotypically masculine traits (i.e., endorsement of stereotypically masculine traits minus endorsement of stereotypically feminine traits).

Competence Evaluations Predicting Additional Forms of Treatment and Related Reactions

Willingness to Support the Employee. Primary analyses demonstrated that managers' biased competence evaluations (favoring the male employee) translated into biased treatment of the employee – specifically, a tendency to provide more distinctive treatment, which is grounded in and effectively conveys the distinct level of competence, value and worth seen in the employee. These analyses tested whether a similar bias emerged in managers' willingness to provide supportive treatment. Such treatment does not entail provision of any prestigious

or high-status opportunities (e.g., taking on new managerial responsibilities) but rather entails provision of more general assistance or support (e.g., additional resources, accommodation) so to help the employee 'keep up' with their colleagues.

Analyses paralleled those described in the main text, and results demonstrated a similar effect: biased competence evaluations (favoring the male employee) translated into a greater willingness to provide support and assistance to the employee, but only among managers who believed women no longer experience discrimination. Specifically, while evaluations of employee competence were critical overall to predicting managers' willingness to provide support ($B = .39$, $p < .001$) these competence evaluations were themselves systematically biased among those who believed women no longer face bias (favoring the male employee; condition*bias-belief, $B = .22$, $p < .001$), which translated into bias in their willingness to support the employee. In other words, there was a significant indirect effect of employee (target) gender on willingness to provide support, but only among those who believed women no longer face bias: indirect effect = .18 [.06, .36]. Among those who rejected this belief, target gender had no significant bearing on managers' willingness to offer support to the employee (indirect effect = -.09 [-.22, .00]).

Analysis examining two items intended to reflect an *un*willingness to invest in or support the employee showed the same general effect. Among those who believed women no longer face bias, their gender-biased competence evaluations translated into a greater likelihood of 'divesting' from the employee (more likely to suggest the employee switch to part-time work, find a job elsewhere): indirect effect = -.11 [-.27, -.01]. By comparison, among those who rejected this belief, employee gender played no significant role (indirect effect = .05 [.00, .18]).

Importantly, while these results demonstrated a similar effect to those described in the main text, the magnitude of the effect was noticeably smaller – half the size, in fact. This indicates that while (biased) competence evaluations are relevant to understanding managers' (biased) tendency to offer general support to the employee, their competence perceptions play a less critical role here than they do in explaining their tendency to treat the employee in distinctly positive ways (e.g., offering unique managerial opportunities). More generally this suggests that distinctive- and supportive forms of treatment are both conceptually separable and different in their most central antecedents.

Differential Weighting of Performance Information. Some evidence[53] suggests that when individuals express subtle biases – for instance, in evaluating a female (vs. male) job applicant who has a mix of qualities and drawbacks – they may proceed to strategically de/emphasize certain information about the applicant so to justify their biased evaluation (e.g., suggesting the female applicant's 'strong points' are not actually that strong or important to consider). To test this idea we examined whether expressions of biased competence evaluations (among those who believed women no longer face bias) predicted a subsequent differential evaluation/weighting of the employee's performance information (in a way that would seemingly substantiate their biased competence evaluation; via PROCESS Model 7). Results demonstrated just that. Competence evaluations predicted managers' tendency to emphasize positive performance information overall (B = 6.74, p < .001), but with these competence evaluations biased among those who believed women do not face bias (condition*bias-belief, B = .22, p < .001) this yielded a bias in how they evaluated/weighed performance information. Specifically, results demonstrated that it was only among those who endorsed this belief that the target employee's gender had an indirect effect on managers' tendency to emphasize positive performance information, favoring the male employee, B = 3.13 [1.31, 5.49]. By comparison, among those who rejected this belief, the target employee's gender had no bearing on how managers evaluated/weighed their performance information (B = -1.47 [-3.41, 0.06]). Notably, follow-up tests (PROCESS Model 1) showed no direct moderated effect of target gender on managers' weighting of performance information (condition*bias-belief, B = .90, p = .38) suggesting that the tendency to differentially weigh performance information is strongly premised on a manager's initial expression of a biased competence evaluation.

Perceived Fit and Career Potential. An initial set of analyses, paralleling primary analyses examining competence perceptions (PROCESS Model 1), tested for broad evidence of gender bias in managers' perceptions of the employee's: (i) overall 'fit' for this profession, and (ii) career potential – akin to the bias evident in competence perceptions, namely among those who believed women no longer face discrimination. Regarding fit, analyses revealed no differences in perceptions of the male versus female employee's general fit to the profession (condition*bias-belief, B = .10, p = .23), either among managers who rejected (B = -.22, p = .23) or endorsed (B = .09, p = .60) this belief. Regarding career potential however, analyses showed a trending effect (condition*bias-belief, B = .14, p = .10) such that those who rejected this belief showed no difference in their perceptions of the

fe/male employee's career potential (*B* = -.05, *p* = .44) but managers who endorsed it viewed the male employee as having more potential than the otherwise identical female employee (*B* = .38, *p* = .04).

Additionally, to test whether perceptions of an employee's competence (and any bias therein) might explain how managers perceived the employee in terms of fit for the profession and overall career potential, follow-up analyses tested whether there was an indirect effect of employee gender on perceived fit / potential, via perceived competence. Analyses paralleled those described in main text (PROCESS Model 7, using the standardized composite measure of competence). Results showed that evaluations of employee competence were indeed critical overall to predicting how managers perceived the employee in terms of their fit (*B* = .74, *p* < .001) and career potential (*B* = .77, *p* < .001). Moreover, given that these competence evaluations were consistently biased (favoring the male employee) among those who believed women no longer face discrimination, this ultimately translated into biased evaluations of fit and career potential. In other words, there was an indirect effect of employee gender on perceived fit and career potential, but only among managers who endorsed this belief (fit: indirect effect = .34 [.15, .56], potential: indirect effect = .35 [.15, .58]). Among those who rejected this belief, employee gender had no bearing on perceived fit or potential (fit: indirect effect = -.16 [-.35, .00], potential: indirect effect = -.16 [-.35, .01]).

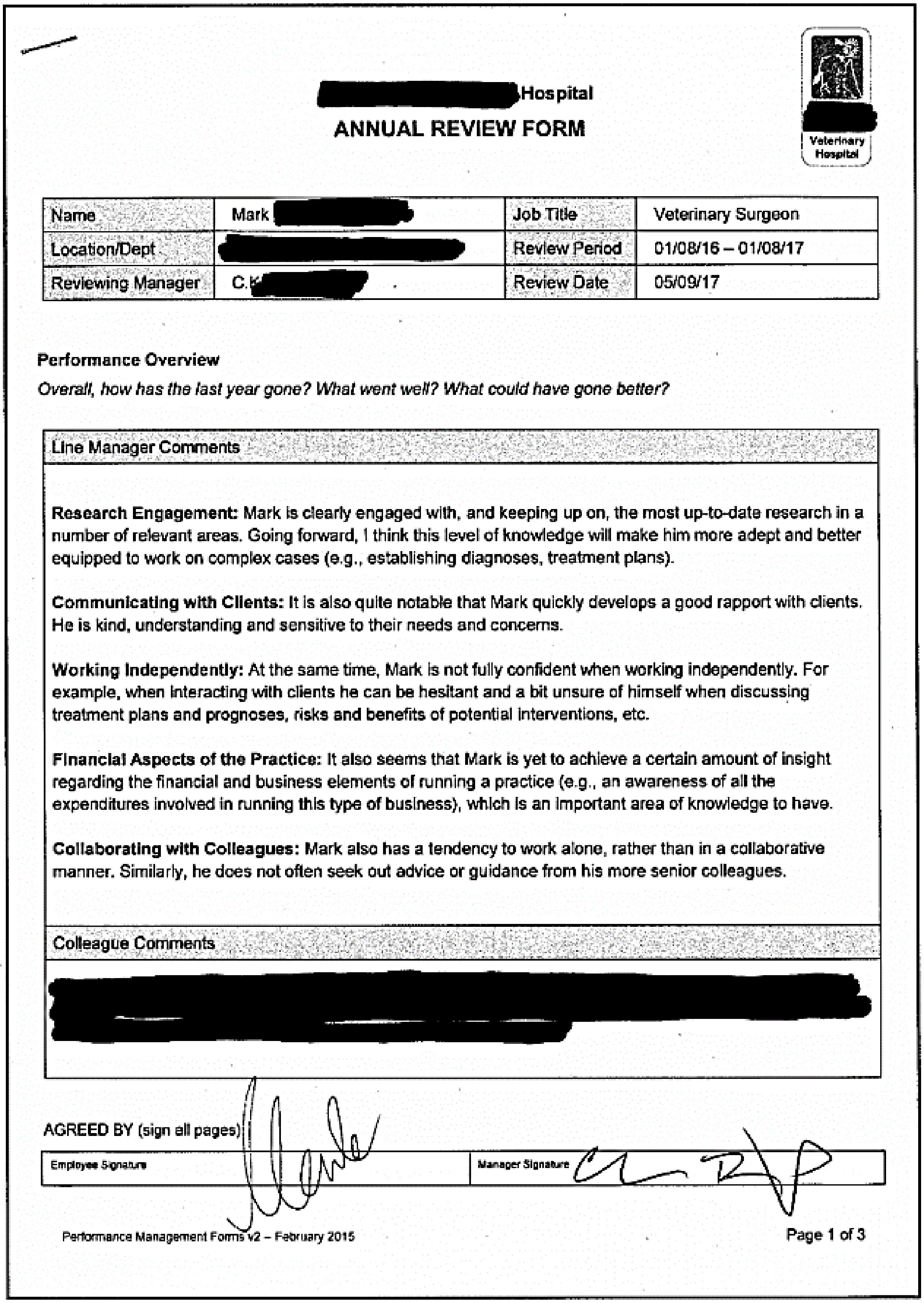

[redacted] Hospital

ANNUAL REVIEW FORM

[redacted] Veterinary Hospital

Name	Mark [redacted]	Job Title	Veterinary Surgeon
Location/Dept	[redacted]	Review Period	01/08/16 – 01/08/17
Reviewing Manager	C.[redacted]	Review Date	05/09/17

Performance Overview

Overall, how has the last year gone? What went well? What could have gone better?

Line Manager Comments

Research Engagement: Mark is clearly engaged with, and keeping up on, the most up-to-date research in a number of relevant areas. Going forward, I think this level of knowledge will make him more adept and better equipped to work on complex cases (e.g., establishing diagnoses, treatment plans).

Communicating with Clients: It is also quite notable that Mark quickly develops a good rapport with clients. He is kind, understanding and sensitive to their needs and concerns.

Working Independently: At the same time, Mark is not fully confident when working independently. For example, when interacting with clients he can be hesitant and a bit unsure of himself when discussing treatment plans and prognoses, risks and benefits of potential interventions, etc.

Financial Aspects of the Practice: It also seems that Mark is yet to achieve a certain amount of insight regarding the financial and business elements of running a practice (e.g., an awareness of all the expenditures involved in running this type of business), which is an important area of knowledge to have.

Collaborating with Colleagues: Mark also has a tendency to work alone, rather than in a collaborative manner. Similarly, he does not often seek out advice or guidance from his more senior colleagues.

Colleague Comments

[redacted]

AGREED BY (sign all pages)

Employee Signature	Manager Signature

Performance Management Forms v2 – February 2015

Page 1 of 3

Fig. S1a. Study 2 experimental stimuli, depicting the annual performance review of a veterinary surgeon. There were two versions, differing only in the purported name of the vet ("Mark," "Elizabeth") and the corresponding pronouns used (male, female). The male version is presented here.

Vet Performance & Assessment

In this section, we would like to get your insights on
vet performance and assessment, as well as training and development.

On the next page you will see a copy of a recent performance review, provided by a BVA-affiliated clinic (mixed practice). It is of a veterinary surgeon.

He graduated from veterinary school just over a year ago, and joined this practice shortly after graduating. The following comes from his first annual performance review, which was conducted shortly after his completion of the RCVS PDP General Competences.

On subsequent pages, we will ask you to share your perspectives about this vet's performance review. For confidentiality, we have redacted all identifying information. For brevity, we have included just a portion of the review.

On the next page, please take some time to read over the performance review.

Fig. S1b. Study 2 cover story (male version) shown to managers just before the experimental stimuli. Managers randomly assigned to the female version of the performance review saw the same text but with female pronouns used throughout.

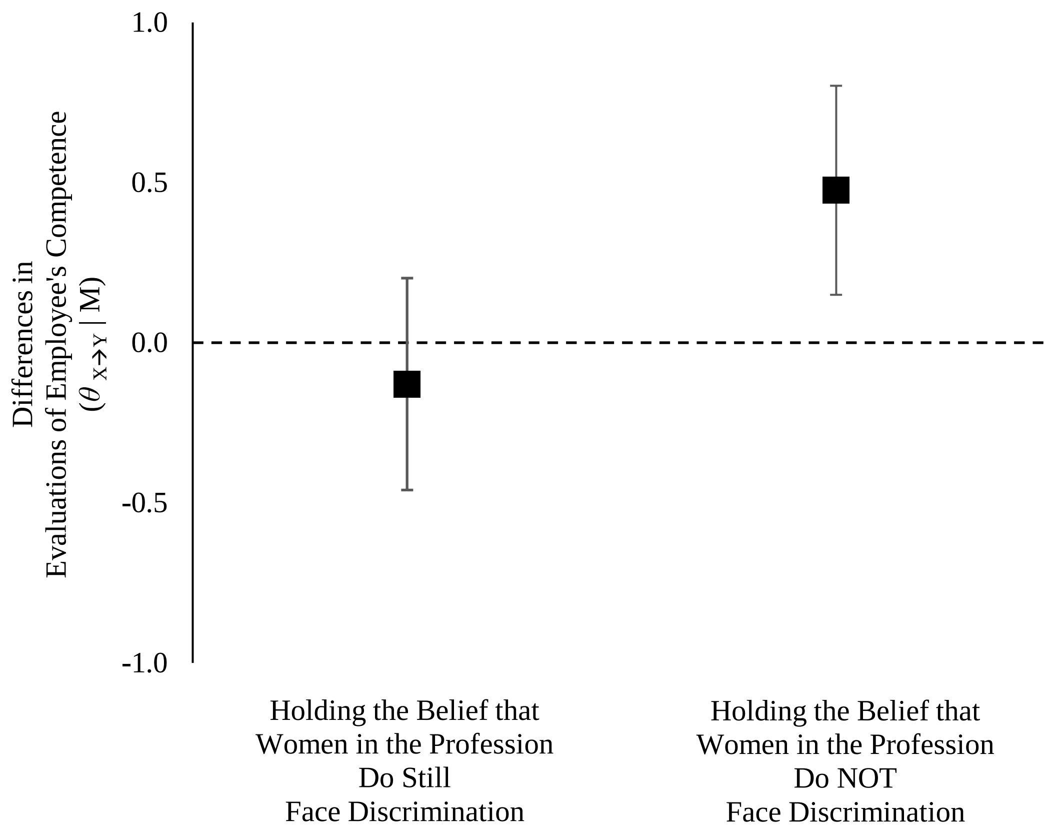

Fig. S2. Differences in evaluations of employee competence (general measure), by the purported gender of the target employee and managers' beliefs about whether women in their profession still face discrimination. (*n* = 236) Plotted values represent the estimated mean difference in competence evaluations (Y) for the male versus female employee (X) by managers' beliefs (M, at +/- 1 *SD* with covariates at their sample means; $\theta_{X \rightarrow Y} | M$). Larger absolute values indicate a greater difference in mean competence evaluations for the male versus female employee. Positive values indicate the male employee was evaluated as more competent than the otherwise identical female employee. Error bars represent 95% confidence intervals; those encompassing the zero point on the y-axis indicate that there was no significant difference in evaluations of the male versus female employee. These mean differences correspond to the following values: 'Holding the Belief that Women in the Profession Do Still Face Discrimination,' $\theta_{X \rightarrow Y} = -.13$, $SE = .17$, $p = .44$ [-.46, .20]; 'Holding the Belief that Women in the Profession Do NOT Face Discrimination,' $\theta_{X \rightarrow Y} = .48$, $SE = .17$, $p = .004$ [.15, .80]. For a depiction of the estimated means, see Fig. 2 in the main text.

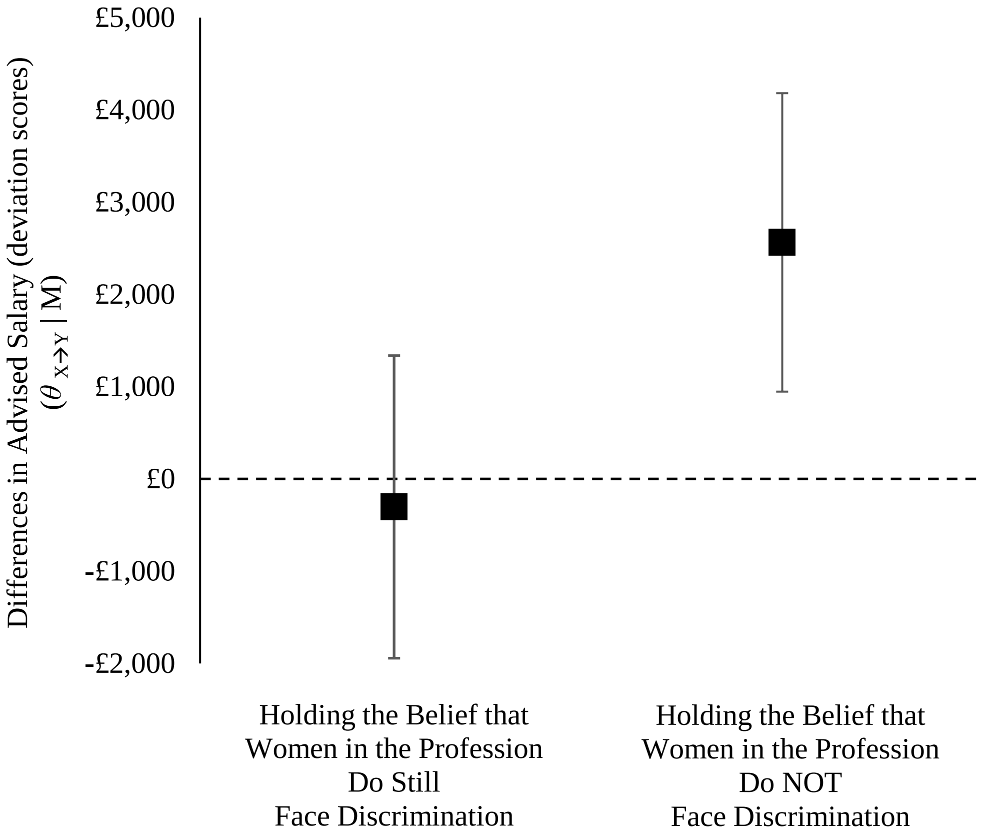

Fig. S3. Differences in advised salary for the target employee, by the purported gender of the employee and managers' beliefs about whether women in their profession still face discrimination. (n = 229) Plotted values represent the estimated mean difference in advised salaries (Y, accounting for individual differences in base salary rates; for more detail, see Fig 2 caption) for the male versus female employee (X) by managers' beliefs (M, at +/-1 *SD* with covariates at their sample means; $\theta_{X \rightarrow Y} \mid M$). Larger absolute values indicate a greater mean difference in advised salary for the male versus female employee. Positive values indicate that managers advised the male employee receive a higher salary than the otherwise identical female employee. Error bars represent 95% confidence intervals; those encompassing the zero point on the y-axis indicate that there was no significant mean difference in advised salaries for the male versus female employee. These mean differences correspond to the following values: 'Holding the Belief that Women in the Profession Do Still Face Discrimination,' $\theta_{X \rightarrow Y}$ = -£303.07, *SE* = £832.00, p = .72 [-£1,942.79, £1,336.65]; 'Holding the Belief that Women in the Profession Do NOT Face Discrimination,' $\theta_{X \rightarrow Y}$ = £2,564.23, *SE* = £820.71, p = .002 [£946.78, £4,181.69]. For a depiction of the advised salaries, see Fig. 3 in the main text.

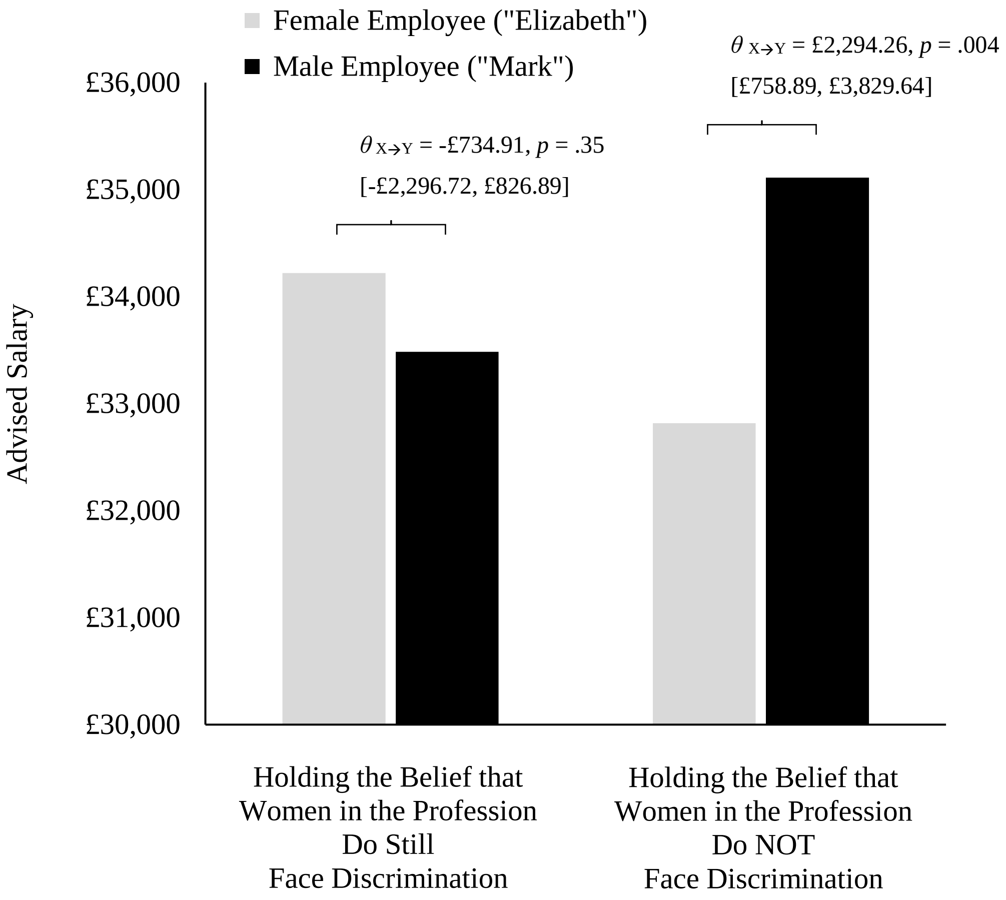

Fig. S4. Managers' advised salary (raw) for the target employee, by the purported gender of the employee and managers' beliefs about whether women in their profession still face discrimination. (n = 229) To account for individual differences in base salary rates, managers reported the typical salary in their practice for employees with similar experience as the target, and this was entered as an additional covariate. Among managers who believe discrimination against women is no longer an issue, they advised the male employee receive a higher salary than the otherwise identical female employee. Analyses probed the interaction (by managers' beliefs) at +/- 1 *SD*. These values correspond to a general endorsement / rejection of these beliefs. For ease of interpretation and because the values represent categorically distinct beliefs, they are presented as bars (estimated means at +/- 1 *SD* with covariates at their sample means). The differences in means correspond to the following values: 'Holding the Belief that Women in the Profession Do Still Face Discrimination,' $\theta_{X \rightarrow Y}$ = -£734.91, *SE* = £792.45, p = .35 [-£2,296.72, £826.89]; 'Holding the Belief that Women in the Profession Do NOT Face Discrimination,' $\theta_{X \rightarrow Y}$ = £2,294.26, *SE* = £779.04, p = .004 [£758.89, £3,829.64]. For an analogous depiction with the above confidence intervals (around the conditional effect of target gender, $\theta_{X \rightarrow Y} \mid M$) see Fig. S5.

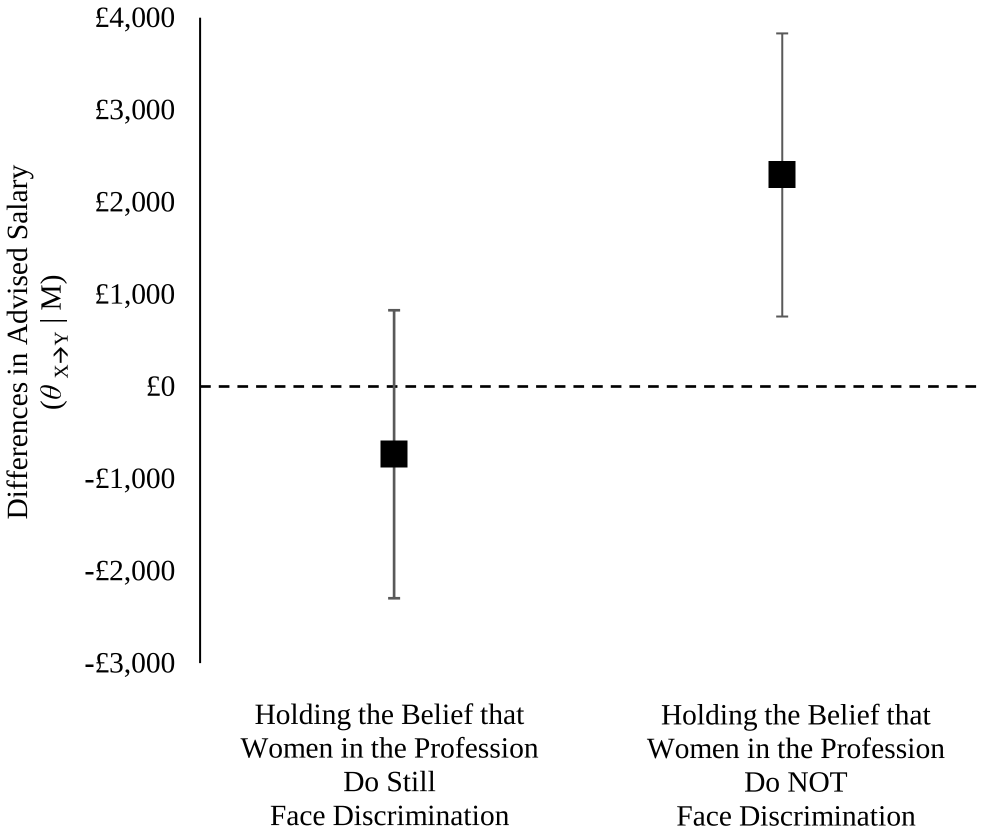

Fig. S5. Differences in advised salary (raw) for the target employee, by the purported gender of the target employee and managers' beliefs about whether women in their profession still face discrimination. (n = 229) Plotted values represent the estimated mean difference in advised salaries (Y, accounting for individual differences in base salary rates [entered as an additional covariate]) for the male versus female employee (X) by managers' beliefs (M, at +/- 1 *SD* with covariates at their sample means; $\theta_{X \rightarrow Y}$ | M). Larger absolute values indicate a greater mean difference in advised salary for the male versus female employee. Positive values indicate that managers advised the male employee receive a higher salary than the otherwise identical female employee. Error bars represent 95% confidence intervals; those encompassing the zero point on the y-axis indicate that there was no significant mean difference in advised salaries for the male versus female employee. These mean differences correspond to the following values: 'Holding the Belief that Women in the Profession Do Still Face Discrimination,' $\theta_{X \rightarrow Y}$ = -£303.07, *SE* = £832.00, p = .72 [-£1,942.79, £1,336.65]; 'Holding the Belief that Women in the Profession Do NOT Face Discrimination,' $\theta_{X \rightarrow Y}$ = £2,564.23, *SE* = £820.71, p = .002 [£946.78, £4,181.69]. For a depiction of the advised salaries, see Fig. S4.

REFERENCES AND NOTES

1. NSF National Center for Science and Engineering Statistics, *Women, Minorities, and Persons with Disabilities in Science and Engineering* (NSF National Center for Science and Engineering Statistics, 2019); https://ncses.nsf.gov/pubs/nsf19304/digest.
2. U.S. Department of Labor, Women's Bureau, *Employment and Earnings by Occupation 2018* (U.S. Department of Labor, 2018); https://www.dol.gov/agencies/wb/data/occupations.
3. B. Murphy, *These Medical Specialties Have the Biggest Gender Imbalances* (American Medical Association, 2019); https://ama-assn.org/residents-students/specialty-profiles/these-medical-specialties-have-biggest-gender-imbalances.
4. A. J. Koch, S. D. D'Mello, P. R. Sackett, A meta-analysis of gender stereotypes and bias in experimental simulations of employment decision making. *J. Appl. Psychol.* **100**, 128–161 (2015).
5. C. A. Moss-Racusin, J. F. Dovidio, V. L. Brescoll, M. J. Graham, J. Handelsman, Science faculty's subtle gender biases favor male students. *Proc. Natl. Acad. Sci. U.S.A.* **109**, 16474–16479 (2012).
6. B. Welle, M. E. Heilman, *Formal and Informal Discrimination Against Women at Work: The Role of Gender Stereotypes* (The Center for Public Leadership Working Papers 18, Harvard Univ. Press, 2005).
7. S. E. Holleran, J. Whitehead, T. Schmader, M. R. Mehl, Talking shop and shooting the breeze: A study of workplace conversation and job disengagement among STEM faculty. *Soc. Psychol. Personal. Sci.* **2**, 65–71 (2011).
8. N. DiTomaso, C. Post, D. R. Smith, G. F. Farris, R. Cordero, Effects of structural position on allocation and evaluation decisions for scientists and engineers in industrial R&D. *Adm. Sci. Q.* **52**, 175–207 (2007).
9. S. Atir, M. J. Ferguson, How gender determines the way we speak about professionals. *Proc. Natl. Acad. Sci. U.S.A.* **115**, 7278–7283 (2018).
10. R. Jagsi, K. A. Griffith, A. Stewart, D. Sambuco, R. De Castro, P. A. Ubel, Gender differences in the salaries of physician researchers. *JAMA* **307**, 2410–2417 (2012).
11. P. L. Carr, A. Raj, S. E. Kaplan, N. Terrin, J. L. Breeze, K. M. Freund, Gender differences in academic medicine: Retention, rank, and leadership comparisons from the national faculty survey. *Acad. Med. J. Assoc. Am. Med. Coll.* **93**, 1694–1699 (2018).

12. R. van Veelen, B. Derks, M. D. Endedijk, Double trouble: How being outnumbered and negatively stereotyped threatens career outcomes of women in STEM. *Front. Psychol.* **10**, 150 (2019).
13. C. Seron, S. Silbey, E. Cech, B. Rubineau, "I am Not a Feminist, but. . .": Hegemony of a meritocratic ideology and the limits of critique among women in engineering. *Work Occup.* **45**, 131–167 (2018).
14. G. Huang, "Seeking women: 70+ companies that have set gender diversity targets," *Forbes* (2017); https://forbes.com/sites/georgenehuang/2017/02/14/seeking-women-40-companies-that-have-set-gender-diversity-targets/.
15. C. W. Greider, J. M. Sheltzer, N. C. Cantalupo, W. B. Copeland, N. Dasgupta, N. Hopkins, J. M. Jansen, L. Joshua-Tor, Gary S. Mc Dowell, J. L. Metcalf, B. A. McLaughlin, A. Olivarius, E. K. O'Shea, J. L. Raymond, D. Ruebain, J. A. Steitz, B. Stillman, S. M. Tilghman, V. Valian, L. Villa-Komaroff, J. Y. Wong, Increasing gender diversity in the STEM research workforce. *Science* **366**, 692–695 (2019).
16. National Academies of Sciences, Engineering, and Medicine, *Promising Practices for Addressing the Underrepresentation of Women in Science, Engineering, and Medicine: Opening Doors* (The National Academies Press, 2020).
17. J. Huang, A. Krivkovich, I. Starikova, L. Yee, D. Zanoschi, *Women in the Workplace 2019* (Mckinsey & Company, 2019); https://mckinsey.com/~/media/McKinsey/Featured%20Insights/Gender%20Equality/Women%20in%20the%20Workplace%202019/Women-in-the-workplace-2019.ashx.
18. R. M. Kanter, Some effects of proportions on group life, in *The Gender Gap in Psychotherapy: Social Realities and Psychological Processes*, P. P. Rieker, E. Carmen, Eds. (Springer US, 1984), pp. 53–78.
19. N. Ellemers Gender stereotypes. *Annu. Rev. Psychol.* **69**, 275–298 (2018).
20. C. L. Williams The glass escalator: Hidden advantages for men in the "female" professions. *Soc. Probl.* **39**, 253–267 (1992).
21. C. L. Ridgeway, Interaction and the conservation of gender inequality: Considering employment. *Am. Sociol. Rev.* **62**, 218–235 (1997).
22. B. A. Nosek, M. R. Banaji, A. G. Greenwald, Harvesting implicit group attitudes and beliefs from a demonstration web site. *Group Dyn. Theory Res. Pract.* **6**, 101–115 (2002).

23. B. A. Nosek, M. R. Banaji, A. G. Greenwald, Math = male, me = female, therefore math ≠ me. *J. Pers. Soc. Psychol.* **83**, 44–59 (2002).
24. F. L. Smyth, B. A. Nosek, On the gender–science stereotypes held by scientists: Explicit accord with gender-ratios, implicit accord with scientific identity. *Front. Psychol.* **6**, 415 (2015).
25. D. I. Miller, A. H. Eagly, M. C. Linn Women's representation in science predicts national gender-science stereotypes: Evidence from 66 nations. *J. Educ. Psychol.* **107**, 631–644 (2015).
26. K. L. Milkman, M. Akinola, D. Chugh, What happens before? A field experiment exploring how pay and representation differentially shape bias on the pathway into organizations. *J. Appl. Psychol.* **100**, 1678–1712 (2015).
27. D. Z. Grunspan, S. L. Eddy, S. E. Brownell, B. L. Wiggins, A. J. Crowe, S. M. Goodreau, Males under-estimate academic performance of their female peers in undergraduate biology classrooms. *PLOS ONE* **11**, e0148405 (2016).
28. U.S. Department of Education, National Center for Education Statistics, *Digest of Education Statistics: Bachelor's, Master's, and Doctor's Degrees Conferred by Postsecondary Institutions, by Sex of Student and Discipline Division, 2016–2017* (U.S. Department of Education, 2018); https://nces.ed.gov/programs/digest/d18/tables/dt18_318.30.asp.
29. UK Higher Education Statistics Agency, *Higher Education Qualifications Obtained by Subject Area and Sex 2012/13 to 2016/17* (UK Higher Education Statistics Agency, 2018); https://hesa.ac.uk/data-and-analysis/sfr247/figure-18.
30. L. S. Wilton, D. T. Sanchez, M. M. Unzueta, C. Kaiser, N. Caluori, In good company: When gender diversity boosts a company's reputation. *Psychol. Women Q.* **43**, 59–72 (2019).
31. J. K. Swim, K. J. Aikin, W. S. Hall, B. A. Hunter, Sexism and racism: Old-fashioned and modern prejudices. *J. Pers. Soc. Psychol.* **68**, 199–214 (1995).
32. J. K. Swim, L. L. Cohen, Overt, covert, and subtle sexism: A comparison between the attitudes toward women and modern sexism scales. *Psychol. Women Q.* **21**, 103–118 (1997).
33. E. L. Uhlmann, G. L. Cohen, "I think it, therefore it's true": Effects of self-perceived objectivity on hiring discrimination. *Organ. Behav. Hum. Decis. Process.* **104**, 207–223 (2007).

34. B. Monin, D. T. Miller, Moral credentials and the expression of prejudice. *J. Pers. Soc. Psychol.* **81**, 33–43 (2001).
35. I. Régner, C. Thinus-Blanc, A. Netter, T. Schmader, P. Huguet, Committees with implicit biases promote fewer women when they do not believe gender bias exists. *Nat. Hum. Behav.* **3**, 1171–1179 (2019).
36. H. Kelly, "Google commits $150 million to diversity," *CNNMoney* (2015); https://money.cnn.com/2015/05/06/technology/google-diversity-plan/index.html.
37. The Royal College of Veterinary Surgeons, *RCVS Facts 2017: Facts and Figures from the Royal College of Veterinary Surgeons* (The Royal College of Veterinary Surgeons, 2018), pp. 1–26; https://rcvs.org.uk/news-and-views/publications/rcvs-facts-2017/.
38. "Veterinary women: Past, present and future," *Veterinary Woman* (2015); https://veterinarywoman.co.uk/2015/02/veterinary-women-past-present-and-future/.
39. The Royal College of Veterinary Surgeons, *RCVS Facts 2008: The Annual Report of the Royal College of Veterinary Surgeons: Part 2* (The Royal College of Veterinary Surgeons, 2008), pp. 1–23; https://rcvs.org.uk/news-and-views/publications/rcvs-facts-2008/.
40. F. Danbold, Y. J. Huo, Men's defense of their prototypicality undermines the success of women in STEM initiatives. *J. Exp. Soc. Psychol.* **72**, 57–66 (2017).
41. J. F. Dovidio, S. L. Gaertner, Aversive racism and selection decisions: 1989 and 1999. *Psychol. Sci.* **11**, 315–319 (2000).
42. A. F. Hayes, *Introduction to Mediation, Moderation, and Conditional Process Analysis: A Regression-Based Approach* (Guilford Press, 2013).
43. A. Waters, Gender pay gap exists across the profession. *Vet. Rec.* **182**, 92–93 (2018).
44. J. T. Jost, M. R. Banaji, The role of stereotyping in system-justification and the production of false consciousness. *Br. J. Soc. Psychol.* **33**, 1–27 (1994).
45. C. S. Crandall, A. Eshleman, A justification-suppression model of the expression and experience of prejudice. *Psychol. Bull.* **129**, 414–446 (2003).
46. S. J. Ceci, W. M. Williams, S. M. Barnett Women's underrepresentation in science: Sociocultural and biological considerations. *Psychol. Bull.* **135**, 218–261 (2009).
47. C. T. Begeny, Y. J. Huo, Is it always good to feel valued? The psychological benefits and costs of higher perceived status in one's ethnic minority group. *Group process. Intergroup Relat.* **21**, 193–213 (2018).

48. C. T. Begeny, Y. J. Huo, When identity hurts: How positive intragroup experiences can yield negative mental health implications for ethnic and sexual minorities. *Eur. J. Soc. Psychol.* **47**, 803–817 (2017).
49. J. Cohen, *Statistical Power Analysis for the Behavioral Sciences* (L. Erlbaum Associates, 1988).
50. UK Gender Pay Gap Service, *Gender Pay Gap Reporting: Make Your Calculations* (UK Gender Pay Gap Service, 2019); https://gov.uk/guidance/gender-pay-gap-reporting-make-your-calculations.
51. C. L. Hoyt, J. L. Burnette, Gender bias in leader evaluations: Merging implicit theories and role congruity perspectives. *Pers. Soc. Psychol. Bull.* **39**, 1306–1319 (2013).
52. UK Office for National Statistics, *Top 100 Baby Names in England and Wales: Historical Data* (UK Office for National Statistics, 2014); https://www.ons.gov.uk/peoplepopulationandcommunity/birthsdeathsandmarriages/livebirths/datasets/babynamesenglandandwalestop100babynameshistoricaldata.
53. G. Hodson, J. F. Dovidio, S. L. Gaertner, Processes in racial discrimination: Differential weighting of conflicting information. *Pers. Soc. Psychol. Bull.* **28**, 460–471 (2002).
54. P. Glick, S. T. Fiske, The ambivalent sexism inventory: Differentiating hostile and benevolent sexism. *J. Pers. Soc. Psychol.* **70**, 491–512 (1996).
55. M. K. Ryan, S. A. Haslam, M. D. Hersby, R. Bongiorno, Think crisis–think female: The glass cliff and contextual variation in the think manager–think male stereotype. *J. Appl. Psychol.* **96**, 470–484 (2011).
56. M. E. Heilman, Gender stereotypes and workplace bias. *Res. Organ. Behav.* **32**, 113–135 (2012).
